# Spatio-Temporal Representativeness of Air Quality Monitoring Stations in Mexico City: Implications for Public Health

**DOI:** 10.3389/fpubh.2020.536174

**Published:** 2021-01-12

**Authors:** Karol Baca-López, Cristóbal Fresno, Jesús Espinal-Enríquez, Mireya Martínez-García, Miguel Angel Camacho-López, Miriam V. Flores-Merino, Enrique Hernández-Lemus

**Affiliations:** ^1^School of Medicine, Autonomous University of the State of Mexico, Toluca de Lerdo, Mexico; ^2^Computational Genomics Division, National Institute of Genomic Medicine, Mexico City, Mexico; ^3^Technological Development Office, National Institute of Genomic Medicine, Mexico City, Mexico; ^4^Centro de Ciencias de la Complejidad, Universidad Nacional Autónoma de México, Mexico City, Mexico; ^5^Sociomedical Research Unit, National Institute of Cardiology ‘Ignacio Chávez’, Mexico City, Mexico; ^6^School of Chemistry, Autonomous University of The State of Mexico, Toluca de Lerdo, Mexico

**Keywords:** public policy, air pollution, missing data, geo-temporal analysis, semivariogram

## Abstract

Assessment of the air quality in metropolitan areas is a major challenge in environmental sciences. Issues related include the distribution of monitoring stations, their spatial range, or missing information. In Mexico City, stations have been located spanning the entire Metropolitan zone for pollutants, such as CO, NO_2_, O_3_, SO_2_, PM_2.5_, PM_10_, NO, NO_*x*_, and PM_*CO*_. A fundamental question is whether the number and location of such stations are adequate to optimally cover the city. By analyzing spatio-temporal correlations for pollutant measurements, we evaluated the distribution and performance of monitoring stations in Mexico City from 2009 to 2018. Based on our analysis, air quality evaluation of those contaminants is adequate to cover the 16 boroughs of Mexico City, with the exception of SO_2_, since its spatial range is shorter than the one needed to cover the whole surface of the city. We observed that NO and NO_*x*_ concentrations must be taken into account since their long-range dispersion may have relevant consequences for public health. With this approach, we may be able to propose policy based on systematic criteria to locate new monitoring stations.

## 1. Introduction

As population density, mobility, and industrial activity keep growing at an accelerated rate, air pollution has gained the attention of policy makers in urban and metropolitan areas. There is a common concern in highly polluted cities regarding the increasing mortality associated with chronic and acute diseases whose effects may be aggravated due to exposure to air contaminants (1–3).

It is well-known that different diseases or health-related effects depend on both the exposure time and concentration levels (1). As evidence suggests, not only long periods of exposure can be damaging, but exposure to high levels in short periods—even a few hours—may have an immediate negative impact (4, 5).

**Figure 1:**
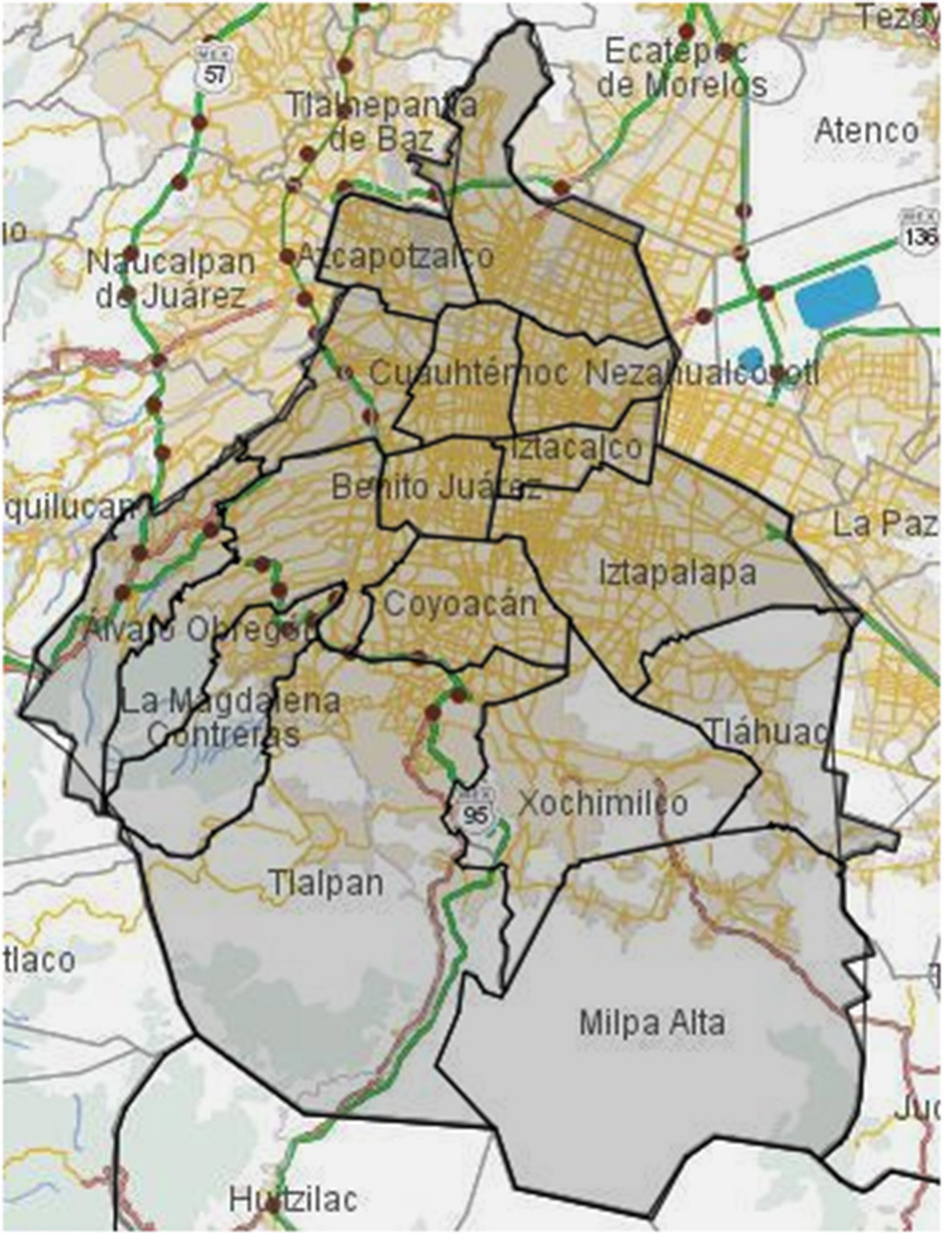
Map of land use in Mexico City. In this figure, the 16 boroughs of the Mexico capital city are delimited by black lines. Additionally, the streets of the metropolitan area of Mexico City are also depicted. It can be observed that the southern region of this city (Tlalpan, Milpa Alta, part of Xochimilco, and Tlahuac boroughs) is less dense in terms of urban environment. Map obtained from INEGI.

Mexico City (Figure 1), as many other metropolis worldwide, has implemented strategies for urban planning, transportation, and regulations of industrial activity to reduce contaminant emissions (6). As an example, the Metrobus transport system started operating in the year 2005 as an emission reduction strategy. By comparing CO, NO_*x*_, PM_10_, and SO_2_ measurements before and after the Metrobus operations, a reduction ranging from 5 to 9% for different contaminants in city areas was observed (7). Another example is driving restriction policy in Mexico City, which was originally set only for weekdays. In an attempt to improve results, the program extended this restriction to Saturdays without meeting the expected results of reducing emission by almost 15% (8).

So far, these efforts were not successful as planned. On the contrary, pollution levels have not decreased, which is noticeable from the continuous environmental alerts throughout the years. Some contaminants, such as particulate matter PM_10_ vary seasonally; however, some regulations may be effective for this type of pollutant, some may not be useful for others (9).

In this regard, public policies will only be effective if they rely on the proper identification of pollution sources, the understanding of the dispersion dynamics, and the adequate measurement of relevant variables.

### 1.1. The Relevance of Pollution Monitoring and Assessment

Determining the number and distribution of air quality monitoring stations depends on the area to be covered, traffic, spatial variability due to land use, influence of meteorological variables (temperature, wind speed, and ultraviolet radiation), and dispersion dynamics of each pollutant (10–12).

Environmental policy planning needs reliable methods to assess the risk level associated with exposure to chemical and other noxious agents. This latter can be made by direct and indirect measurements of pollutants with epidemiological and toxicological dimensions.

Direct approaches require the estimation of the incidence of undesired effects by considering individual exposures to contaminants. Environmental hazard of this kind often relies on the analysis of spatial data collected by environmental surveys (13).

Monitoring networks have two main purposes. First, by measuring spatial and temporal trends of pollutant concentrations, they provide air quality estimations to determine whether the population is exposed to dangerous levels or not. In addition, with the use of social-demographic, land use related variables and meteorological data, simulation models can guide to better decision-making procedures.

Second, once implemented, the effectiveness of public policies and regulations can be evaluated by analyzing changes in pollution levels that are caused merely by the imposed regulations. Thus, the development of a monitoring system is a critical component of public health policy making to decrease toxic emission and eventually prevent population from adverse contaminant effects.

A relevant emerging concept is environmental health surveillance. For this concept, the quality and completeness of information has been found variable, depending on individual hazards or exposures, even in well-developed public health surveillance systems, such as the one in Canada (13).

The Mexico City Ministry of Environment (Secretaría del Medio Ambiente, SEDEMA) is responsible for the establishment of measuring procedures, data gathering, and reporting air quality levels (14). The estimations are based on the measurement of carbon monoxide (CO), nitrogen dioxide (NO_2_), ground level ozone (O_3_), sulfur dioxide (SO_2_), small fine particulate matter (PM_2.5_ and PM_10_), nitrogen oxide (NO), other nitrogen oxides (NO_*x*_), and coarse particulate matter (PM_*CO*_).

Although Mexico City's monitoring network meets international standards, it fails to have complete records for all the contaminants. In some cases, continuous monitoring stations stopped functioning due to technical reasons and maintenance, whereas others just stopped operating and in some cases, measurements were not registered while they were still active.

The appropriate functioning of such monitoring stations is extremely relevant to public health issues (15). It is known, for instance, that ozone and particulate matter (PM_2.5_) levels have been closely associated with a number of adverse health effects that may lead to premature mortality (16). Such effects are particularly relevant in the context of urban environments (17).

### 1.2. Evaluation of Health Impact and Monitoring Stations

The progressive incorporation of information sampling and retrieval technologies and the use of geographic information systems (GIS) to analyze the data have become a central tenet of Health Impact Assessment (HIA) programs. The way to analyze the data however is shifting from merely transaction reports to the use of advanced analytics, such as the ones used in business intelligence and data science.

Latin American countries have developed specialized programs to make use of GIS and computational intelligence to improve their HIA programs. Studies, such as the ALBA, GeoSur, or in the case of Mexico, the Global Environmental Outlook (GEO) are aiming in this direction (13).

GEO has indeed developed its own strategy within the “geotext” framework in order to use spatial analysis to provide policy makers (and even the public) with enhanced information resources, however these resources are just as good as the information they are based on (18, 19).

In the case of air pollution monitoring stations, the WHO has actually advanced some guidelines as to what standards are desirable for the data sources to be useful in the context of HIA programs (20).

Mexico City is doing partially well according to these standards; however, our results have shown that there are things that need improvement, in particular taking into account the size and urban characteristics of the metropolitan area of Mexico City as large urban areas pose particular environmental challenges (21).

It has been discussed that increased risks created by urban development include unhealthy conditions, which may arise from unplanned settlements or rapidly growing urban environments, environmental pollution by over-concentration of waste and other pollutants, and overcrowding, among others (22).

### 1.3. The Question of Spatial Representativeness

Determiniation of the spatial representativeness of background monitoring stations from concentration measurements of air pollutants, has been a matter of intense research (23–27). It has been shown that the size and shape of representative areas differ between pollutants and measured locations, and representative areas may range from 220 to 4,500 km^2^ (24).

To improve the assessment of coverage estimation in the case of a limited number of stations, detailed pollutant concentration maps at pedestrian level have been used (27). In this example, for Pamplona, Spain, the authors found that ~18% of the entire area is well-represented, as most of the residential areas are included. This result states that it is possible to assess the covered area by air quality networks integrated by a limited number of stations for a small city (23 km^2^).

The most complete study on the spatial representativeness of monitoring sites is the JCR Technical Report developed by the Forum for Air Quality Modeling in Europe (FAIRMODE) (28, 29). The aim was to perform an inter-comparison of 25 assessment methods from 14 different countries based on a literature review of scientific journals and technical documentation.

The outcomes of the above-mentioned study were established to define spatial representativeness and to propose standard methodological procedures for European country members. The different methodologies can be categorized according to their assessment criteria, such as modeling, measurements, proxies, station classifications, and annual concentrations. The outputs from these studies are presented as delimited areas or size parameters.

In order to have an adequate assessment of the effectiveness of those monitoring networks, the city's spatial heterogeneities should be taken into account.

To estimate the concentrations at unmeasured locations, interpolation methods, such as land-use regression (LUR), inverse distance weighting (IDW), or kriging, use historical data from monitoring stations and other monitoring procedures (30, 31). These estimations are mainly used for health risk assessment. Prediction of high values and trends helps to guide decisions both, locally and at citywide levels.

Recently, kriging geo-statistical approach has been used to analyze spatial representativeness from NO_2_ preliminary concentrations in urban areas (26, 32). The kriging methodology for spatio-temporal interpolation is based on the covariance data structure on spatial or spatio-temporal level. To achieve that task, the empirical semivariogram is modeled with a parsimonious covariance structure, through the use of different kernel functions, to determine the spatial and temporal correlation range.

### 1.4. Intervention Policy and Assessment

The development of analytical approaches to determine and assess environmental pollution data with the best spatio-temporal granularity is key in the design and implementation of proper intervention policy, for example, regarding urbanization process, over-population, personal monitors, indoor environments, vehicle fleet, peak hours, and green areas in the city, among others (33).

The *PAHO Regional Plan on Urban Air Quality and Health 2000–2009* has proposed efficient systems for air pollution health impact monitoring. These must include periodic surveillance of morbidity and mortality associated with air pollution, risk assessment, effective information systems, and reliable estimation of social costs related to air pollution.

In this regard, research designs, such as the one advanced here will help to address some of the main concerns included in the PAHO plan and also allow us to comply with the agreements on other initiatives, such as the *Air Management Information Systems* (AMIS).

To this end, Mexico (as a country) has developed a nationwide air quality monitoring program (the *Sistema Nacional de Información de la Calidad del Aire*, SINAICA https://sinaica.inecc.gob.mx/). It is worth noticing that the flagship implementation of SINAICA has been indeed the metropolitan area of Mexico City.

The information derived from the SINAICA program (in particular the one constituted in the PROAIRE initiative) has already allowed the country to develop general policies to improve the air quality (the PROAIRE strategies for emission reduction).

The PROAIRE website includes a quite comprehensive repository of resources useful for research and policy making that can be found at http://www.aire.cdmx.gob.mx/descargas/publicaciones/flippingbook/proaire-2011-2020-anexos/.

Research efforts along these lines, although admittedly far from complete, have allowed to implement public health policy to lower the negative health impact on air pollution. Take, for instance, the case of ozone, whose high levels are known to affect human health, in particular that of vulnerable or over exposed groups, such as athletes, outdoor workers, asthmatics and people with respiratory illnesses, and children.

It has been reported that by implementing some of the recommendations in the PROAIRE initiative, average ozone levels in the Metropolitan area of Mexico City diminished from almost 0.18 parts per million (ppm) in 1991 to around 0.1 ppm in 2007. These levels have remained below (34). It is expected that such a decrease in the ozone levels would also decrease respiratory illness incidence.

It is, however, complex to determine the real impact of such measures, although HIA programs have pointed out that by implementing appropriate policies up to 33,084 ozone-related deaths may have been prevented in Mexico City during the period of 2000–2020 (35).

Another study in three of the largest cities in the Americas (Mexico City, São Paulo and New York) reported similar results. In Cifuentes et al. (36), it was mentioned that during the period of 2000–2020, up to 64,000 premature deaths could be prevented, just by reducing the levels of ozone and particulate matters in around 10%.

### 1.5. Scope and Outline of This Work

In this work, we present a novel methodology based on the use of spatial and temporal variogram ranges modeling to estimate monitoring stations representativeness. This methodology does not require estimation of pollutant concentration (full interpolation procedure).

This work aims to show the temporal evolution of spatial representativeness of monitoring stations in Mexico City, one of the most complex networks and metropolitan areas worldwide. Additionally, temporal representativeness is shown, which is not the case for most of these studies. We explore these spatial coverage and temporal dependence on measurement for all pollutants currently reported in Mexico City.

In brief, two main questions are addressed here:

- What is the spatial and temporal representativeness of the air quality monitoring network in Mexico City?
- Which is the space/time range within which sample point measurements are correlated with measured values at monitoring stations?

We also discuss about the public health implications of these questions and how can we use this information to provide feedback to health and public policy makers.

## 2. Materials and Methods

### 2.1. Study Area

Mexico City, which belongs to the Valley of Mexico Metropolitan Area (VMMA), is located at 99°21′53.64″ − 98°56′25.08″ West and 19°2′53.52″ − 19°35′34.08″ North. By the year 2015, its total population was 8,985,339 as reported by the National Institute of Statistics and Geography (Instituto Nacional de Estadística y Geografía, INEGI) (37). The polygon shape files of Mexico City were obtained from the National Institute of Statistic and Geography (Instituto Nacional de Estadística y Geografía, INEGI) (37). Hereafter, the geo-spatial data granularity was kept at the 16 available boroughs (municipalities).

### 2.2. Air Pollution Database

The Mexico City Air Quality Monitoring System public database is available from the Aire CDMX website (38). For this study, the required data were accessed using the R package aire.zmvm (39).This database is part of the Automatic Network of Atmospheric Monitoring (RAMA, according to the Spanish acronym of Red Automática de Monitoreo Atmosférico).

The network was established by the Metropolitan Environmental Commission of Mexico City to monitor compliance with ambient air quality standards. The RAMA is part of the Atmospheric Monitoring System (SIMAT, Sistema de Monitoreo Atmosférico), a program responsible for ongoing measurements of the main air pollutants in Mexico City.

SIMAT comprehends 70 monitoring stations distributed along the VMMA, where only 49 of them report contaminant levels. At each monitoring station, hourly contaminant concentrations are available for (i) particulate matter with an aerodynamic diameter of <2.5 and 10 μ*m*, PM_2.5_, and PM_10_, respectively, (ii) carbon monoxide (CO), (iii) ozone (O_3_), (iv) sulfur dioxide (SO_2_), and (v) nitrogen oxides, monoxide, and dioxide (NO_*x*_, NO, and NO_2_).

**Figure 2:**
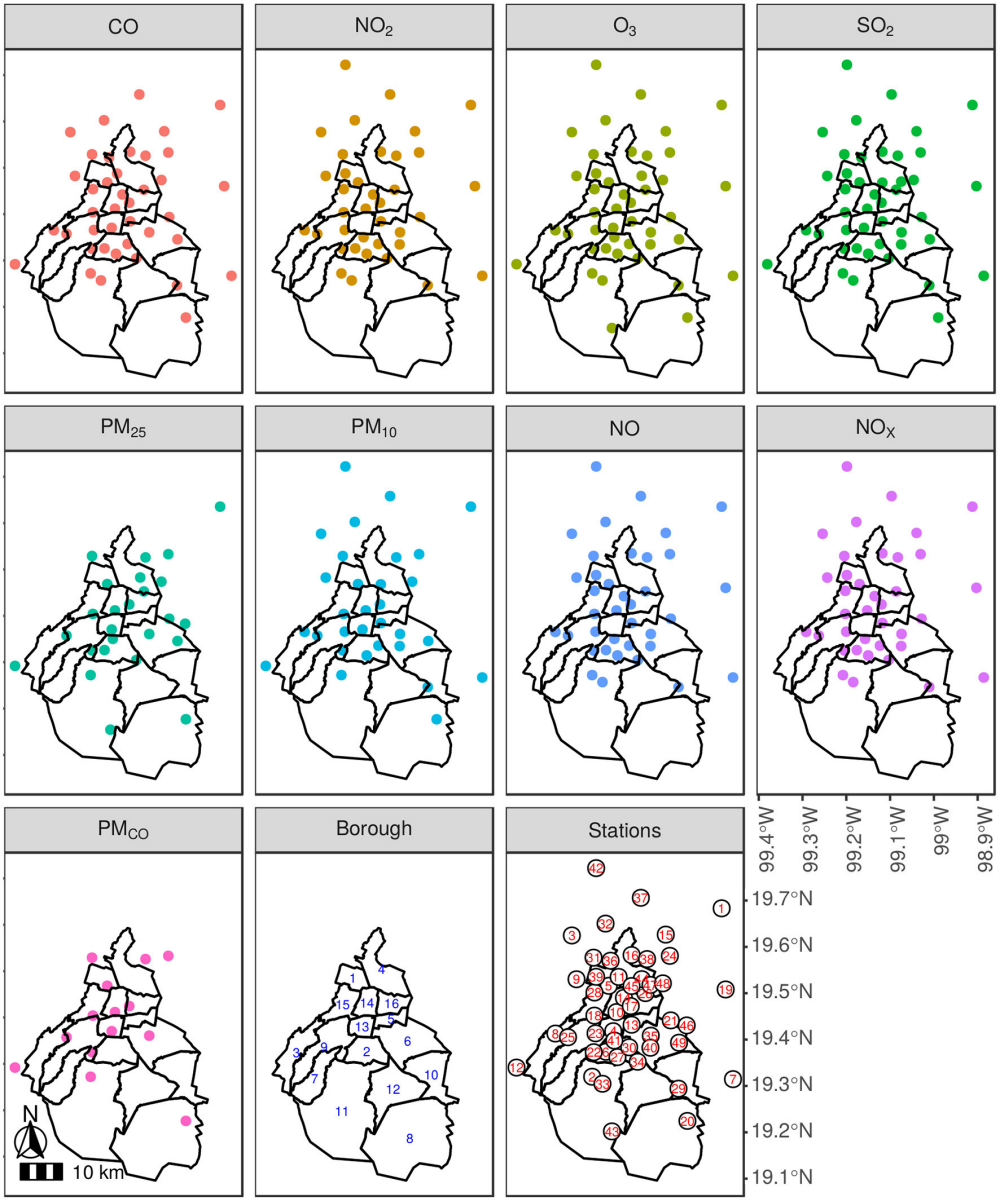
Monitor stations per contaminant in 2019. A borough level description for Mexico City is presented in the different contaminant panels, where the monitor stations are depicted in color dots. The included contaminants are as follows: CO, NO_2_, O_3_, SO_2_, PM_2.5_, PM_10_, NO, NO_*x*_, and PM_*CO*_. In addition, a panel shows borough names: 1: Azcapotzalco, 2: Coyoacán, 3: Cuajimalpa de Morelos, 4: Gustavo A. Madero, 5: Iztacalco, 6: Iztapalapa, 7: La Magdalena Contreras, 8: Milpa Alta, 9: Álvaro Obregón, 10: Tláhuac, 11: Tlalpan, 12: Xochimilco, 13: Benito Juárez, 14: Cuauhtémoc, 15: Miguel Hidalgo, and 16: Venustiano Carranza. A similar idea is used to match the stations location to their corresponding three letter code: 1: ACO, 2: AJM, 3: ATI, 4: BJU, 5: CAM, 6: CCA, 7: CHO, 8: CUA, 9: FAC, 10: HGM, 11: IMP, 12: INN, 13: IZT, 14: LAG, 15: LLA, 16: LPR, 17: MER, 18: MGH, 19: MON, 20: MPA, 21: NEZ, 22: PED, 23: PLA, 24: SAG, 25: SFE, 26: SJA, 27: SUR, 28: TAC, 29: TAH, 30: TAX, 31: TLA, 32: TLI, 33: TPN, 34: UAX, 35: UIZ, 36: VAL, 37: VIF, 38: XAL, 39: AZC, 40: CES, 41: COY, 42: CUT, 43: AJU, 44: GAM, 45: LVI, 46: PER, 47: ARA, 48: FAR, and 49: SAC. Note that for contaminants, such as PM_*CO*_ and PM_2.5_ almost all monitor stations are inside Mexico City, whereas the rest of them include several outside the city.

Naturally, all the monitoring stations do not collect all types of contaminants. Additionally, there exist missing records due to service maintenance or other incidents. The time period used in this work is from 2009 to 2018, when possible. Location of monitor stations for each pollutant can be observed in Figure 2. Complete monitor stations data can be found in Supplementary Table 1.

### 2.3. Spatio-Temporal Statistics

**Figure 3:**
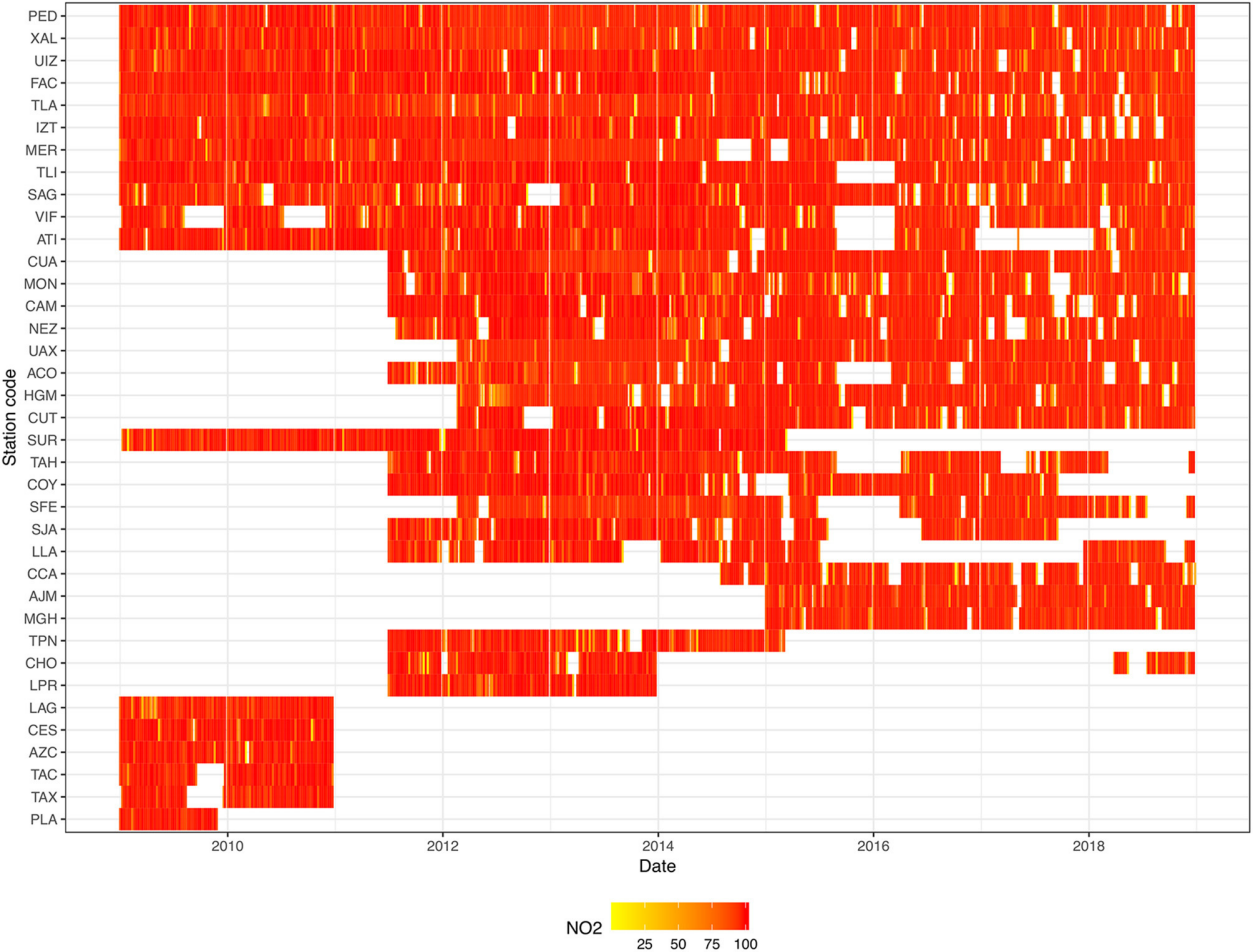
NO_2_ monitor stations representativeness. The tile plot represents data completeness, on a weekly basis, for each monitoring station (three letter code). The color bar indicates the percentage of complete data. Interestingly, there exist some time gaps, whether the stations were not available yet, lost contaminant track, or ended their monitoring life. However, in most of the reported periods, records are almost complete (in red). See Supplementary Table 1 for monitor stations full description.

The collected data were explored to get a clear picture of the pollutant monitor stations representativeness in Mexico City. Hourly contaminant data were summarized by their average into a week time-basis, if more than 5 days were available containing 17-h records or more. Data were plotted for each pollutant. Figure 3 shows data for nitrogen dioxide (NO_2_). The rest of pollutant data can be found in Supplementary Figures 1–8.

In this work, we used the semivariogram to estimate the degree of spatial and temporal dependence between measurements for the different air pollutants. In brief, the semivariogram provides a description of how measurements vary across distance, time or both, as it measures the degree of spatial correlation of a random variable *Z*(*x*), *Z*(*t*), or *Z*(*x, t*), respectively. In particular, for the unidimensional spatial component *Z*(*x*), the experimental semivariogram $\hat{\gamma }\left(h\right)$ that varies with distance *h* is written in equation (1):

(1) $$\begin{array}{l} \hat{\gamma }\left(h\right)=\frac{1}{2N\left(h\right)}\overset{N\left(h\right)}{\underset{i=1}{\sum }}{\left[Z\left(x_{i}+h\right)-Z\left(x_{i}\right)\right]}^{2} \end{array}$$

where *Z*(*x*_*i*_) is the observed value for the *i*th location at coordinate *x*_*i*_, *Z*(*x*_*i*_ + *h*) the observed value at location *x*_*i*_ + *h*, and *N*(*h*) the number of measured points within a distance h. An equivalent representation applies for the temporal component. As the reader can see, the expression presented in equation (1) has no close form, hence, the task here is to find the suitable formulation that best explains the data from one of the following: exponential equation (2), spherical equation (3), or Gaussian equation (4), as described in Chilès and Delfiner (40).

(2) $$\begin{array}{l} {\hat{\gamma }}_{Exponential}\left(h\right)=\left(s-n\right)\left(1-e^{-\frac{h}{ra}}\right)+nH_{\left(0,\infty \right)}\left(h\right) \end{array}$$

(3) $$\begin{array}{l} {\hat{\gamma }}_{Spherical}\left(h\right)=\left(s-n\right)\left(\left(\frac{3h}{2r}-\frac{h^{3}}{2r^{3}}\right)H_{\left(0,r\right)}\left(h\right)+H_{\left[r,\infty \right)}\left(h\right)\right)\\ \;+nH_{\left(0,\infty \right)}\left(h\right) \end{array}$$

(4) $$\begin{array}{l} {\hat{\gamma }}_{Gaussian}\left(h\right)=\left(s-n\right)\left(1-e^{-\frac{h^{2}}{r^{2}a}}\right)+nH_{\left(0,\infty \right)}\left(h\right) \end{array}$$

where *n* is also known as the **nugget**, which can be interpreted as the residual spatial dependence as it is defined as $n=\lim_{h\to 0^{+}}\hat{\gamma }\left(h\right)$, i.e., the y-intercept of the semivariogram $\hat{\gamma }\left(h\right)$ where it is supposed that no correlation with other measurement exists, but the data point itself, when the distance *h* is as close to zero; *s* is a.k.a the **sill**, which is defined as $s=\lim_{h\to \infty^{+}}\hat{\gamma }\left(h\right)$ and it can be interpreted as the limit of the variogram tending to infinity distance; and *r* is a.k.a the **range**, which is the distance where it is satisfied that $\lim_{\forall h>0}\left(\hat{\gamma }\left(r\right)-\hat{\gamma }\left(r+h\right)\right)\to 0$, i.e., the distance where the spatial correlation is lost where the sill levels off, and for a fixed sill model it would be the first time the sill is reached, whereas for a asymptotic sill it would conventionally be the distance where the semivariogram first reaches 95% of the sill; $a=\frac{1}{3}$ as defined in Chilès and Delfiner (40), and *H*_*A*_(*h*) is the unit step Heaviside function, where it is 1 if *h* ∈ *A* and 0 otherwise.

Now, if we move forward toward spatio-temporal correlations, the unidimensional concepts for space (*h*) and time (*u*) need to introduce a covariance structure with its associated semivariogram γ(*h, u*) form for the different implementations included in gstat R package implementation as described in Pebesma (41), Gräler et al. (42), and Baca-Lopez et al. (43) for the following models: *separable* equation (5), *productSum* equation (6 and 7), *metric* equation (8), *sumMetric* equation (9), and *simpleSumMetric* equation (10).

(5) $$\begin{array}{l} \gamma_{separable}\left(h,u\right)=sill\left(\bar{\gamma_{s}}\left(h\right)+\bar{\gamma_{t}}\left(u\right)-\bar{\gamma_{s}}\left(h\right)\bar{\gamma_{t}}\left(u\right)\right) \end{array}$$

(6) $$\begin{array}{l} \gamma_{product\;sum}\left(h,u\right)=\left(k\times sill_{t}+1\right)\gamma_{s}\left(h\right)+\left(k\times sill_{s}+1\right)\gamma_{t}\left(u\right)\\ \;-k\gamma_{s}\left(h\right)\gamma_{t}\left(u\right) \end{array}$$

(7) $$\begin{array}{l} sill_{st}=k\times sill_{s}\times sill_{t}+sill_{s}+sill_{t} \end{array}$$

(8) $$\begin{array}{l} \gamma_{metric}\left(h,u\right)=\gamma_{joint}\left(\sqrt{h^{2}+{\left(\kappa \times u\right)}^{2}}\right) \end{array}$$

(9) $$\begin{array}{l} \gamma_{sum\;metric}\left(h,u\right)=\gamma_{s}\left(h\right)+\gamma_{t}\left(u\right)\\ \;+\gamma_{joint}\left(\sqrt{h^{2}+\left(\kappa \times u^{2}\right)}\right) \end{array}$$

(10) $$\begin{array}{l} \gamma_{simple\;sum\;metric}\left(h,u\right)=n\times H_{h>0\vee u>0}+\gamma_{s}\left(h\right)+\gamma_{t}\left(u\right)\\ \;+\gamma_{joint}\left(\sqrt{h^{2}+\left(\kappa \times u^{2}\right)}\right) \end{array}$$

where for the spatial *s* and time *t* domains, the corresponding variables are *h* and *u*, respectively; the sill parameter has been described explicitly as *sill* to avoid confusion with the space variable *s*; γ_*s*_ and γ_*t*_ are the spatial and temporal semivariograms with their respective standardized versions ${\bar{\gamma }}_{s}$ and ${\bar{\gamma }}_{t}$ with separate nugget effects and (joint) sill of 1; *k* is a positive parameter, i.e., *k* > 0 that satisfies equation (7); κ is the spatio-temporal anisotropy (stAni) correction; *n* is the nugget parameter; and *H*_*A*_(*h*) is the unit step Heaviside function, where it is 1 if *h* ∈ *A* and 0 otherwise.

The initial semivariogram parameter values were obtained from the empirical spatio-temporal pollutant measurements using gstat R package implementation as described in Pebesma (41), Gräler et al. (42), and Baca-Lopez et al. (43). Here, the initials values are computed as follows:

- **Nugget**: It is the median value of the first three empirical variogram matrix row/column means for the spatial or temporal initial guess.
- **Sill**: It is the median value of the last five empirical variogram matrix row/column means for the spatial or temporal initial guess.
- **Range**: The spatial range is one-third of the lagged maximum spatial value and for the temporal case, it corresponds to the maximum value.

In addition, the spatial and temporal anisotropy was estimated using a linear model as specified in the gstat implementation. Finally, the best parsimonious model was found for each contaminant. Briefly, using the initial variogram parameters, different spatial, temporal, or joint semivariogram structures were tested to find the one that best explained the correlated data description, according to the available implementations in gstat (*metric, separable, productSum, sumMetric*, and *simpleSumMetric*) (41, 42).

In this context, the best parameter combination was found testing all possible single, double, or triple semivariogram combinations (exponential, Gaussian, and spherical), where the model selection criterion used was to minimize the weighted mean squared error (see Supplementary Table 3). Finally, the winner spatio-temporal semivariogram structure was used to extract final semivariogram parameters (see Supplementary Table 4).

Last but not least, the integration of both contaminant's monitoring representativeness plots and final spatio-temporal variogram range parameters were used to get a clear picture of Mexico City pollutant radius representativeness according to the time evolution monitor station activity. The spatial correlation range for each pollutant at a particular year was used as a radii to build a circumference around each monitoring station. Thus, the union of circles from all monitoring stations within the network constitute the covered area for a specific pollutant. This procedure was applied to all years of study to show how covered area has changed over time.

## 3. Results

### 3.1. Spatio-Temporal Data Exploration

Monitoring stations geo-localization are depicted in Figure 7, the 16 boroughs of Mexico City. At first sight, the global picture makes it clear that not all the contaminants are acquired for each available station. Second, it seems that for all the contaminants the south of Mexico City (borough numbers 8, 11, and 12) are not as well-represented as its northern counterpart.

This concern is related to rural and urban distribution, where most urban populated boroughs are located to the north of the city. Indeed, some contaminant stations are located outside Mexico City. Pollutants, such as PM_*CO*_ and PM_25_ are almost exclusively collected inside Mexico City, whereas the rest have monitor stations outside the city.

To further explore the data completeness, the monitor station representativeness plots were generated. In Figure 3, the case of NO_2_ is presented for the available stations from the beginning of 2009 until late 2018.

It is clear that there exists block of missing data (in white), where some of them can be tracked down to the monitoring station inauguration (CUA, MON, CAM, NEZ, and so on) in the mid 2011, or until 2015 for AJM and MGH stations.

As a matter of fact, there are no stations whatsoever that had not lost track of NO_2_ at least for some hours (red to yellow cells) or even had stopped working for a time gap of days, weeks, or months. The last case, can be pictured for TLI, VIF, ATI, and ACO to name some stations in the time window including the beginning of 2016.

The last time pattern can be considered as the complete station shut down, as depicted by the block of LAG, CES, AZC, TAC, and TAX, that stopped working at the end of 2010. Fortunately, these stations are not located neither in the same borough nor close each other to leave non-measured areas (see Figure 2). However, this data description level does not represent the extend covered by each monitoring station.

### 3.2. Spatio-Temporal Variogram Estimation

Using the available data, sample spatio-temporal variograms were addressed. These variograms were used to generate the initial guesses values (Supplementary Table 2). Depending on the contaminant, the initial guesses are different for the nugget, range, sill, and stAni. In this context, the nugget is the model intercept attributable to measurement errors or spatial sources of variation at distances smaller than the sampling interval or both.

Interestingly, these sources of variations are negligible for *CO*, in contrast to the wide range of nugget values (0.03 − 178.67). In addition, the correlation extends between measurements, also known as range; in all cases, it is almost the same for all contaminants and last about 12 years for as far as 21.4 km. The value for the variogram when the distance reaches the range, also known as the sill, is as close to the nugget only for *CO* and departs from it at most double its value.

Final covariance model weighted mean square error for all the tested variogram permutations can be found in Supplementary Table 3. It is worth to mention that the lowest error for the different covariance structure methods was the one that included *sumMetric* for *CO*, *NO*_2_, *O*_3_, *NO*, *NO*_*x*_, *PM*_10_, and *PM*_*CO*_ and *simpleSumMetric* for *SO*_2_ and *PM*_2.5_. Within these covariance models, there was no apparent pattern in the winner variogram model permutation (temporal + spatial + joint).

This is a data-driven approach that required to explore the complete permutation grid in order to reach a parsimonious spatio-temporal correlation model. A visual comparison of each winner covariance model and sample variogram can be found in Figure 4 for NO_2_. The rest of variograms for the other pollutants can be found in Supplementary Figures 9–16.

**Figure 4:**
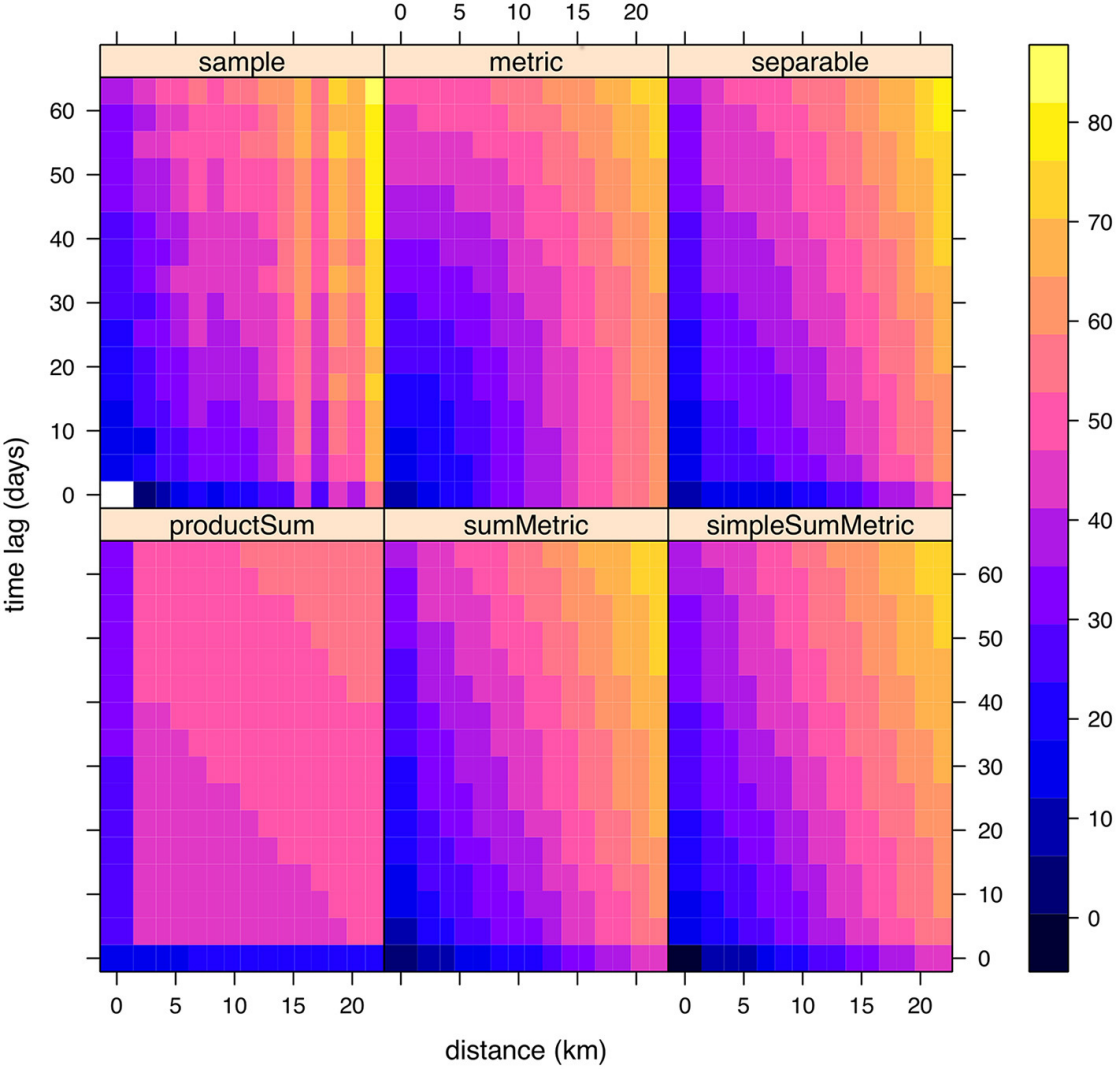
Tested spatio-temporal semivariograms for NO_2_. The 2D-sample semivariogram was obtained from the collected data. In addition, the winner fitted covariance structure models (metric, separable, product sum, sum metric, and simple sum metric) are also presented. In this case, the sum metric structure is the one that outperforms its competitors.

**Figure 5:**
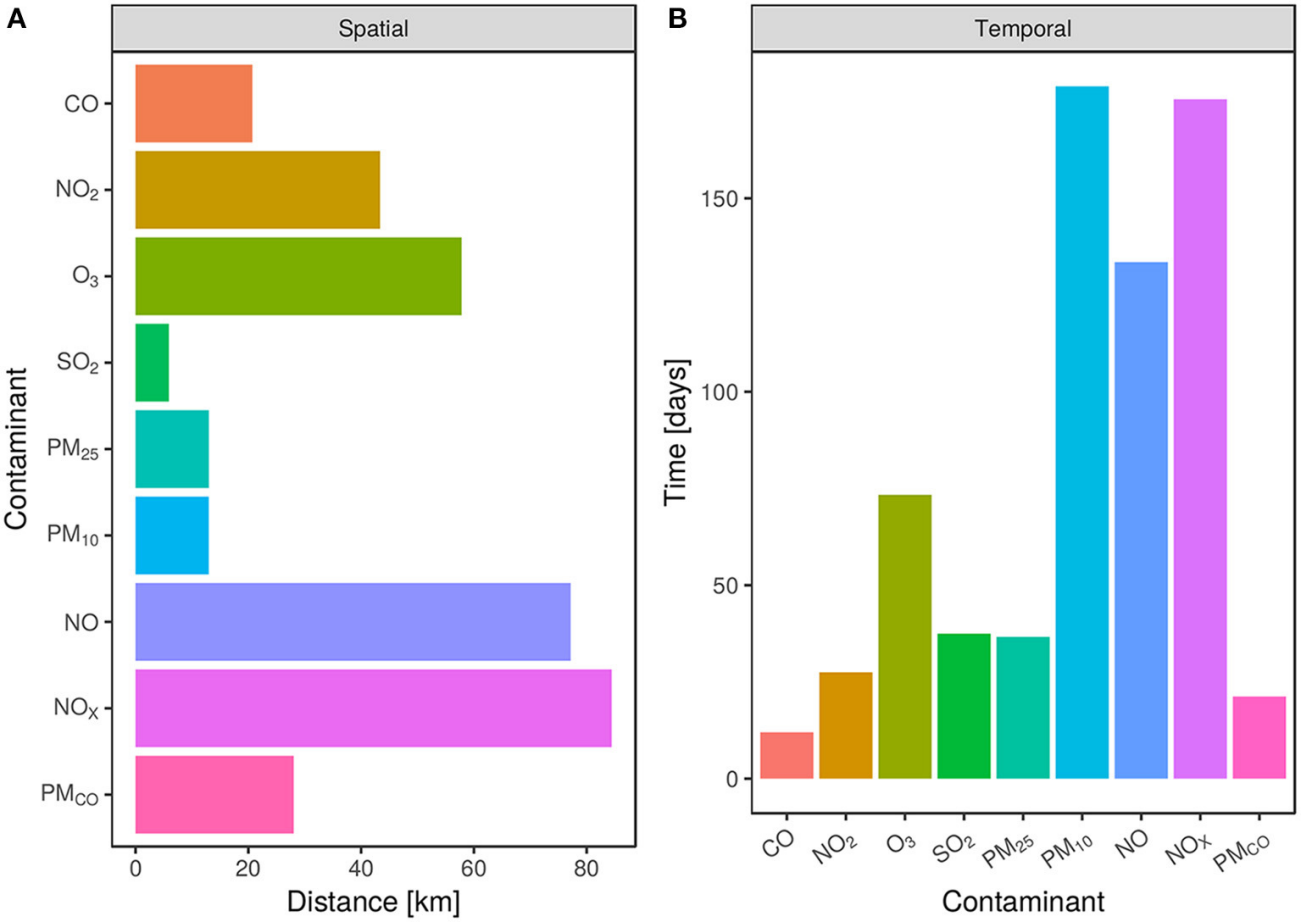
Spatio-temporal range panel. The horizontal or vertical bars stand for the winner covariance structure semivariogram range for each contaminant: CO_2_, O_3_ SO_2_, PM_2.5_, PM_10_, NO, NO_*x*_, and PM_*CO*_. **(A)** Spatial range depicted by horizontal bars. The bigger the bar, the more distance it will be required to find non-correlated data points. **(B)** Temporal range is depicted in vertical bars. Note that the two contaminants with the biggest spatial range (NO and NO_*x*_) are almost the ones with the biggest time range but for PM_10_.

Regarding ranges from winner models, the case of spatial correlation is presented in Figure 5A. The spatial range values measured in kilometer are interpreted as the separation distance between two measured locations, i.e., monitoring stations, that from this value onwards, measurements are no longer correlated.

This is, measured values in a given monitoring station will be correlated with stations located within this range. For instance, NO_*x*_ measurements between monitoring locations have the longest spatial correlation of 84.44 km, followed by NO with 77.15 km. On the contrary, particulate matters PM_2.5_, PM_10_, and SO_2_ have the shortest ranges, 13, 12.98, and 5.92 km, respectively (see Supplementary Table 4).

In the case of temporal correlation, ranges are shown in Figure 5B. PM_10_ has the longest value of ~6 months (178.35 days) followed by NO_*x*_ and NO with ranges of 175.60 and 133.49 days, respectively. These three contaminants differ in great extent in their correlation measurements in comparison to CO, PM_*CO*_, NO_2_, PM_2.5_, S0_2_, and O_3_ with ranges between 12 and 73 days overall (see Supplementary Table 4). This wider time correlation window also presents some implications for environmental control policies, in particular under the scenario of extraordinary events. For instance, abnormal pollution levels may correlate with registers several days apart, hence difficulting emergency decision-making and action taking.

To graphically show the spatial representativeness of each pollutant, we used spatial ranges reported in Figure 4 that were obtained as final parameter values of the variogram models shown in Supplementary Table 4.

For example, for the case of nitrogen dioxide (NO_2_), a spatial range of 43.37 km was obtained from the empirical semivariogram and covariance structure modeling (see Figure 4). Thus, for each monitoring station that measured NO_2_, the center of a circular area with radii 43.37 is matched to the station's location.

To generate a buffer area for NO_2_ to represent the spatial influence for measuring this contaminant, circles traced at each location were joint to define a single border area. This process is performed for NO_2_ in the years 2009, 2012, 2015, and 2018.

As seen in Figure 3, for each year, there is a different number of active monitoring sites. Specifically, in 2009, only 18 sites collected hourly concentrations for NO_2_. In 2012, six additional monitoring stations started collecting data for this contaminant. For the years, 2015 and 2018 the active sites were 26 and 25, respectively. In general, an increasing number of active sites can be seen for all pollutants, starting with the year when measurements began (see Supplementary Figures 1–8).

**Figure 6:**
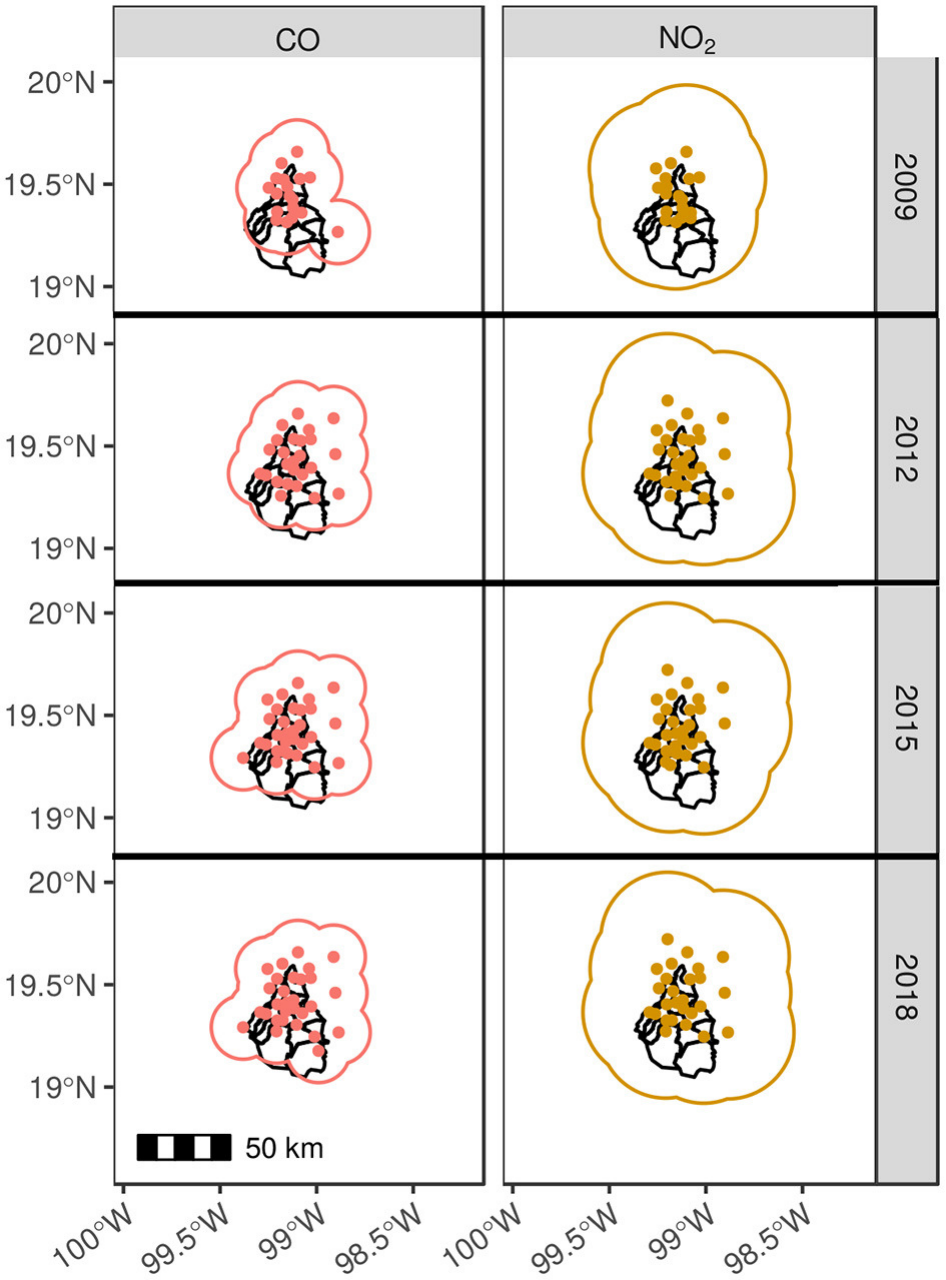
Contaminant representatives radius. The panel is arranged in a matrix style, where the columns correspond to CO and NO_2_ and the rows to years of measurement. In this configuration, each cell represents the Mexico City and the corresponding working monitoring stations. In addition, the contaminant representativeness zone was created by the union of circles with the radius equivalent to the spatial range.

Analogously, using the calculated spatial ranges for CO, NO_2_, O_3_, SO_2_, PM_2.5_, PM_10_ NO, NO_*x*_, and PM_*CO*_, temporal evolution of representativeness areas are shown in Figure 6.

The different contaminants are color coded and displayed as columns, while rows are assigned to selected years. By looking at 2009 year panel, it is noticeable that covered areas between contaminants differ widely. The largest difference in range can be seen for SO_2_ and NO_*x*_ with the smallest and largest ranges, respectively (5.92 and 84.44 km).

The representativeness area for SO_2_ is seen to mostly cover the north part of the city, while the south is not and for the years 2012, 2015, and 2018, similar patterns were obtained.

As expected, although the number of monitored locations increased, there is not a significant increase in the covered area throughout the years due the small range of measurement correlation. This small range depends on the intrinsic physico-chemical properties of SO_2_ and consequently, its diffusion in the atmosphere, as well as due to the complex traits of urban environments.

However, regardless of land use, traffic, population density, and other variables involved for a selected year, it can be seen that for the same locations, the representativeness area for relatively different monitoring networks, these patterns are pollutant dependent.

Similar patterns of an increasing covered area that goes from north to south is observed for CO, PM_2.5_, PM_10_, and PM_*CO*_. It can be seen in the timeline that for these pollutants in the year 2009 (except for PMCO, which was not registered at the time), the southern area was not included in the network but, in the consecutive years this area was extended to almost cover the entire city.

For NO_2_, O_3_, NO, and *NO*_*x*_, regardless of the number of monitoring stations in 2009, because of the large correlation of measurements (ranges), the representativeness area of the network accounts for the whole city and a considerable percentage of the VMMA. Thus, although monitoring stations were added to the network, no significant change is observed for the successive years.

The complete spatio-temporal evolution for these contaminants depicted in Supplementary Figure 17, from years 2009 to 2018, makes clear that regardless of the increase in monitoring stations and their siting location, since 2009 there has been an adequate coverage of the city.

**Figure 7:**
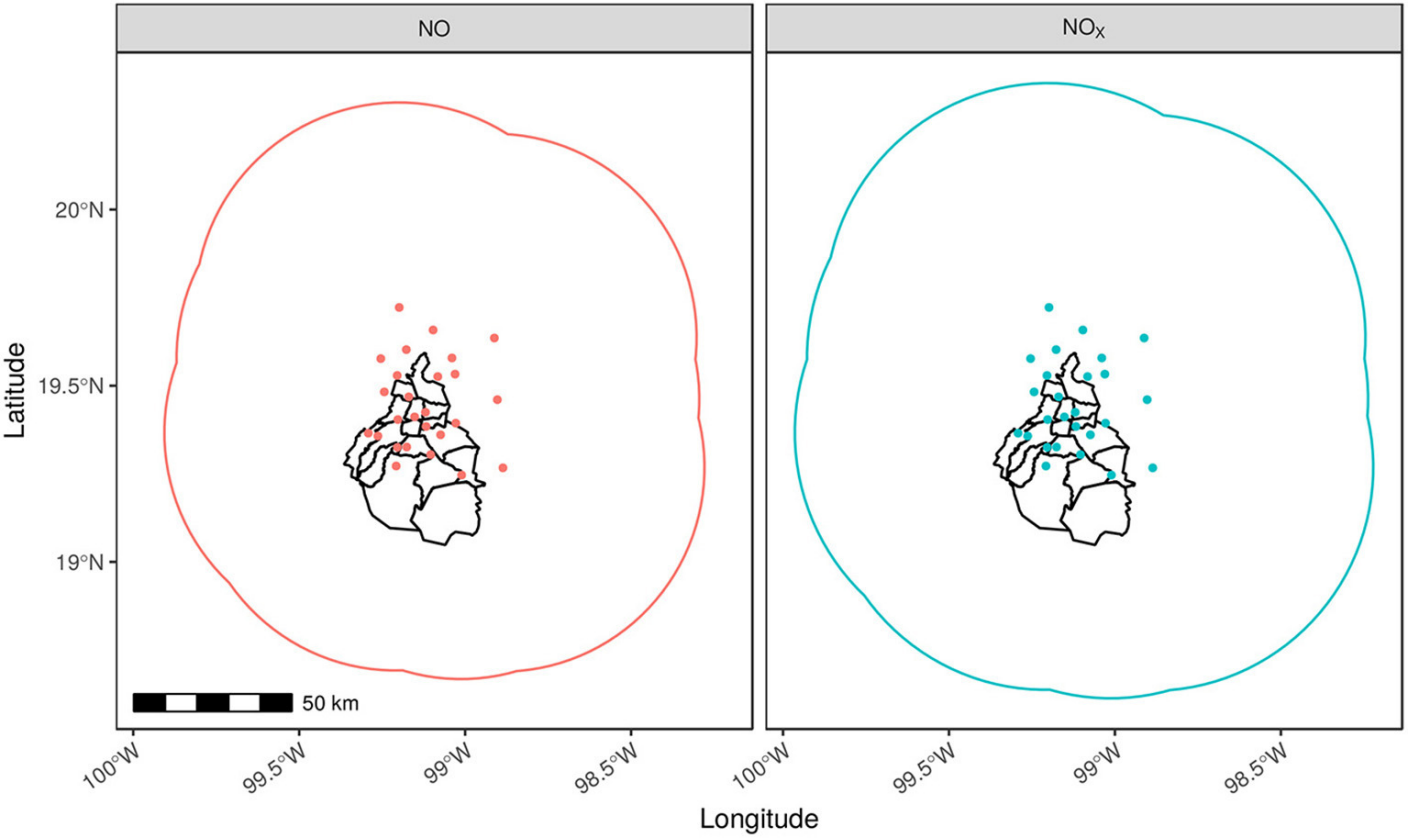
Representativeness areas for NO and NO_*X*_. Covered areas for NO and NO_*x*_ are shown for 2018. Monitoring stations (colored dots) report hourly concentrations of these and other pollutants. In this case, both exhibit long spatial correlations, which in turn indicates a higher correlation between distant monitor stations.

In Figure 6, we present the case of CO and NO_2_. The case of well-represented monitoring networks is shown for NO and NO_*x*_ in Figure 7 as example of pollutants with long spatial correlation, 77.15 and 84.44 km, respectively (see Figure 5). In other words, the amount and selected location for these monitors to construct the network can be considered as effective. It even has shown improvement since throughout the years (see Figure 6).

Another aspect of the NO and NO_*x*_ networks is that their representative area clearly exceeds the city's territory, which is beneficial for both, Mexico City and the VMMA. A relevant issue of this extended coverage is that monitoring stations installed in one of the 16 boroughs in Mexico city are able to capture the influence of pollution from sources outside the city, as these pollutants can eventually move toward the city due to dispersion phenomena.

Additionally, these results allow us to establish neighborhood limits for interpolation purposes. To select the proper number of monitoring stations required to estimate concentration values at unmeasured locations, we can refer to spatial and temporal ranges of correlation to determine which stations have to be included in the analysis.

**Figure 8:**
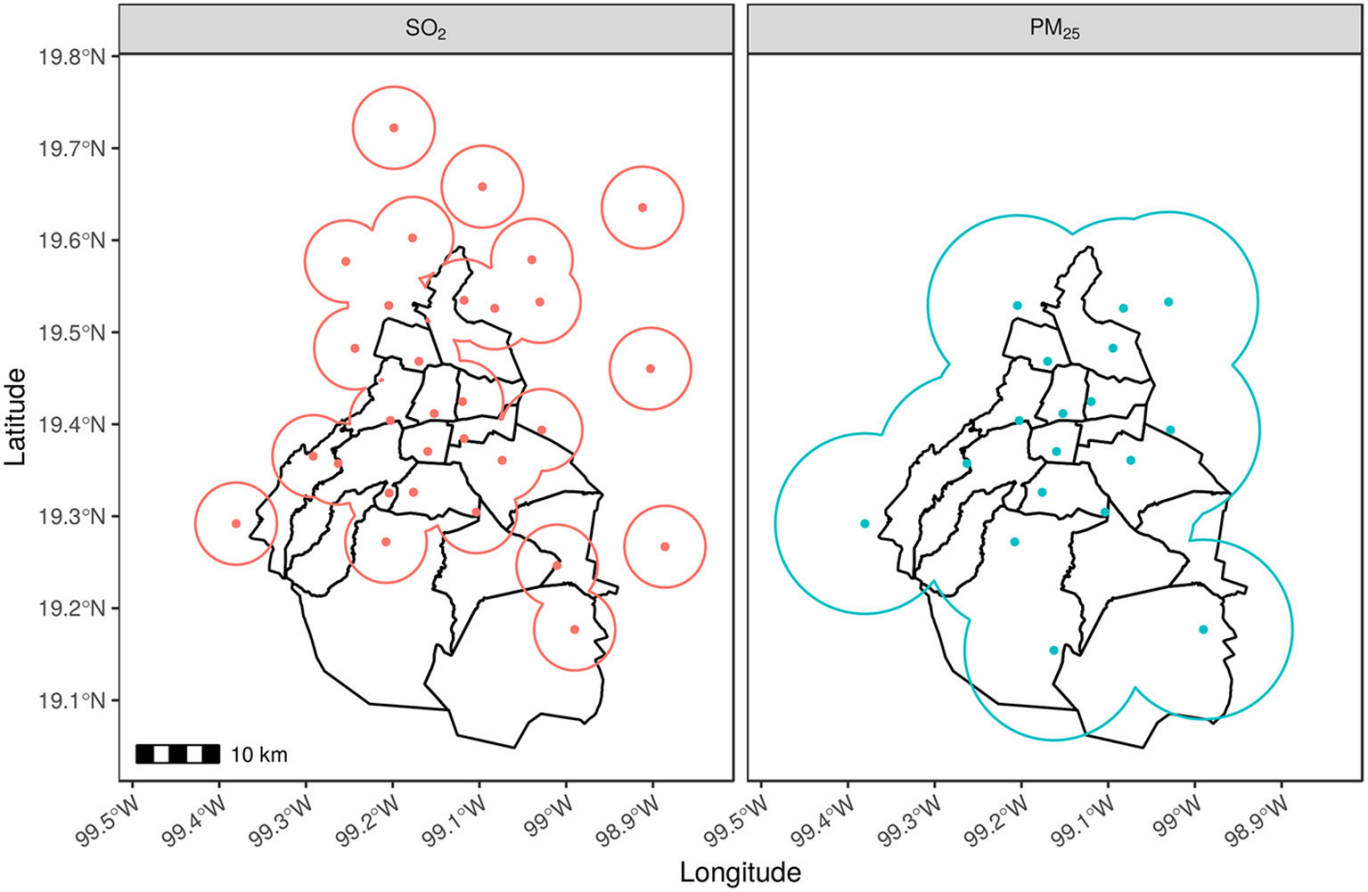
Representativeness areas for SO_2_ and PM_2.5_. A comparison of covered areas for SO_2_ and PM_2.5_ is shown for the year 2018. Monitoring stations (colored dots) report hourly concentrations of these and other pollutants. A different coverage pattern that depends on the spatial range of each pollutant can be seen for SO_2_ and PM_2_.5. In the case of SO_2_, some monitoring stations seem isolated outside the city limits while for PM_2.5_ most of the city surface is well-represented.

An interesting comparison between a well-represented network and one with lack of representativeness is displayed in Figure 8. The full time evolution of the network coverage for SO_2_ and PM_2.5_ is shown in Figure 6, and their status in year 2018 can be seen in Figure 8.

In the case of SO_2_, the central and north parts of the city are almost covered, which is not the case from the center to the south. Additional 15 stations located outside the city, i.e., in the VMMA, are partially connected to the network in the north and four are disconnected.

The current status of PM_2.5_ representation area shows an improvement compared with previous years as seen in Figure 6. For the year 2018 as presented in Figure 8, there is a complete coverage of Mexico City's surface. All areas of individual stations are connected and opposite to SO_2_, this area includes the south.

## 4. Discussion

### 4.1. General Discussion

Air quality assessment is essential for public health, individual, and general population wellness. People's quality of life is strongly determined by the levels of contamination. Hence, to deliver reliable measurements of air pollutants in time and space is of outmost importance. In this work, based on spatio-temporal correlations of monitoring stations of nine air pollutants in Mexico City, we have been able to evaluate whether the location of those stations is adequate to cover the surface of the city.

The analysis showed that the distribution and the number of the monitoring stations is sufficient to evaluate the majority of pollutants, with the exception of sulfur dioxide (SO_2_). The spatial range of monitoring station for SO_2_ is shorter than the other pollutants. The problem does not occur in all zones of the city, and it is constrained to the southern part, as shown in Figures 2, 6, 8. The southern part of Mexico City has a large rural region, and concomitantly, the population density is also short. It is possibly the reason for which there is a limited number of stations in that zone.

Regarding SO_2_, this compound is mainly generated by industrial activity, which is carried into the northern side of Mexico City, and SO_2_ levels in the south are likely due to the dispersion of this pollutant.

With respect to NO and NO_*x*_, and PM_2.5_, whose high levels are crucial in terms of individual and public health, the first two pollutants are well-measured and estimated; however, this is not the case of PM_2_.5 and SO_2_ as the spatial ranges of the monitoring stations for these pollutants are short.

Notwithstanding, in the case of PM_2.5_ monitoring stations, the AJU station (43), which is the southernmost located one, it is able to measure PM_2.5_, and given this, the monitoring stations are able to measure this pollutant cover almost the entire surface of the city. In terms of public policies, an economic and at-hand option to increase the measured surface of SO_2_ stations is to enable the AJU station with SO_2_ capacity.

It is worth to mention that the metropolitan areas are not isolated; contamination could arrive from external places. For instance, during May 2019, Mexico City experimented an unusual environmental challenge due to an elevated concentration of PM_2.5_ and O_3_ (44). Several forest fires have taken place in the Tepozteco National Park and Ajusco National Park, which are both located in the southern border of Mexico City. With this in mind, we state that it is necessary to have also monitoring stations in the periphery of the city to be able to establish, based on spatio-temporal criteria, models to predict contamination indexes and have a better plan for reducing the occurrence of these episodes.

To establish an appropriate methodology to measure air pollutants in time and space, several factors must be taken into account. The correlation between pollutant concentration and health should be carefully evaluated in order to avoid misinterpretations. In what follows, we will discuss some relevant elements that need to be observed.

Depending on the type of pollutant, the residence time in the atmosphere may vary from minutes to weeks. For example, ultra-fine PM (< 0.1μ*m*) remains suspended in the air in the range of minutes to hours. Conversely, PM10 may remain suspended from minutes to hours [Air quality criteria for particulate matter, Washington, DC, US Environmental Protection Agency, 2004 (http://cfpub.epa.gov/ncea/cfm/partmatt.cfm)].

Additionally, photochemical transformations due to sunlight radiation, meteorology, or several other factors should be taken into account in the assessment and the concomitant establishment of public policies.

With this approach, we do not only provide a protocol to measure and estimate areas of representativeness for several pollutants, but also provide suggestions for public policies that are not expensive or logistically complicated. These suggestions may have an impact on the evaluation of the air quality in Mexico City, and hence to help to increase the quality of life of people living in this place.

### 4.2. Extended Dispersion of Emissions: The Case of NO and NO_*x*_

Due to the particular environmental and infrastructural conditions of Mexico City, we have registered a phenomenon of overdispersion (evidenced by wider spatial correlation lengths) of certain pollutants, in particular NO and NO_*x*_ as it was shown in Figure 7. Large amount of nitrogen oxides have been directly related to industrial activity-based von fossil fuels (45).

Due to the specific features of NO and NO_*x*_ in terms of relatively small particle sizes, low aggregation and cluster formations, and other intrinsic physicochemical characteristics, nitric oxide emissions may become over-dispersive under certain atmospheric conditions. This has relevant implications because NO_*x*_ emissions lead to the formation of secondary pollutants, contributing, for instance, to high concentrations of atmospheric ozone (46). Aside from these issues, NO_*x*_ emissions may also contribute to the deposition of NO_3_ creating environmental problems, such as ecosystem acidification.

NO and NO_*x*_ overdispersion also poses additional challenges to regulation and inspection policy. This is so, since in large metropolitan areas, such as Mexico City, extended spatial presence also means that attributing emissions to chemical plants and industries may require deeper inspections and effective scheduling of these (47).

Aside from direct effects of nitric oxides, ozone and particulate matters contribute to important morbidity and mortality. NO, NO_*x*_, and their secondary pollutants may constitute, via widespread exposure, relevant risk-increasing factors to conditions, such as preeclampsia, systemic inflammation, increase in oxidative stress, and cardiovascular events (48, 49). In the case of nitrogen oxides, even causal relationships have been established (50–52). These and other associations with public health concerns will be further discussed in the next subsection.

### 4.3. Public Health Implications

The results just discussed may have important implications in the development of successful HIA programs. HIA programs are aimed at the identification, mitigation, and optimization of the impacts that non-health sector policies may have on public health (53).

Risk quantification used to be mainly based on toxicological or biomedical studies, but more recently the scope of HIAs has broadened to incorporate more general social determinants of health (54).

As it was shown here, using large-scale empirical data from the monitoring network itself, some of the actual challenges have to do with the fact that the radii of coverage are actually different from the various pollutants (see Figure 6).

It is worth mentioning that the regions in that figure correspond to the empirical distribution of air pollutants as given by the characteristic environmental conditions of the metropolitan area of Mexico City and the particular monitoring technology available there.

These facts are indeed matter of current interest, since air pollution in large Latin American cities has become a source of special concerns in recent times. According to a report from the Pan American Health Organization (PAHO) (34), the leading causes of urban air pollution in the Americas are fossil fuels in industry and transportation. The aforementioned report states that in the case of the Mexico City metropolitan area, transportation alone is responsible for some 12% of PM_10_ particles, 30% of PM_2.5_, 5.06% of SO_2_, a staggering 98% of CO, 79% of nitrogen oxides (NO_*x*_), as well as 31% of the volatile organic compounds (34).

As discussed, even if Mexico City has implemented some regulatory systems to reduce air particle concentrations, the results have not been enough to comply with national and international standards. This may be due to the fact that programs approved by policy makers have relied on inadequate air quality measurements.

In these terms, PAHO has been stated that …*there is a clear need for better monitoring systems to analyze trends using more exhaustive, continuous, reliable and complex data and methodologies that are comparable between countries, so that better intervention measures could be adopted to control air pollution …* (34). Our analysis, as presented here, aims to diagnose some aspects of what is missing and what can be improved in terms of the spatio-temporal representativeness of the air quality monitoring stations in the metropolitan area of Mexico City.

Improving our HIA programs and policy is extremely relevant, in particular considering the steady decline in, say PM10 particulate levels, that had been observed from the early 2000s in Mexico City was overturned by a dramatic increase during the years 2008 and 2009. Even if another decrease has been observed since then, we are still lagging to reach the WHO recommended levels. Mexico was, in fact, the country with more deaths due to outdoor air pollution than other countries in the Americas (20,496 in 2012) according to a recent survey (55).

### 4.4. Implications for Intervention Policy

Recalling Figure 6, it is noticeable that intervention policy has indeed improved the quality of monitoring stations for most (but not all) of the pollutants considered.

The metropolitan area of Mexico City has been covered well for ozone levels monitoring since 2009. This, however, was not the case for PM_2.5_ which was poorly covered in the Southside of the city in 2009 and by 2018 has almost complete coverage. A similar case happens to CO levels monitoring which was almost uncovered in 2009 and is well-covered since 2018. Other cases are still worse, more striking in the case of SO_2_ levels, which were poorly covered in the Southside of the city in 2009 and still remains not covered there up to date. This is not to be disregarded since the Southside of Mexico City consists mostly of residential areas with the industrial zones more widely present in the north and east sides of the metropolitan area.

By looking at Figure 6 and Supplementary Figure 1, one can observe that SO_2_ monitoring station facilities have indeed improved in number and effectiveness. However, due to the different coverage features of the stations for the different pollutants, these efforts have been insufficient to date. This is why spatio-temporal representation studies, such as the present are relevant for public policy making.

All the aforementioned facts highlight the relevance of data-driven efforts to improve health impact assessments. Aside from air quality monitoring, there are other data-centered measures that must be implemented, such is the case of exposure evaluation which is indeed essential to calculate risk levels.

A number of epidemiological methodologies have been developed to assess population exposure to air pollutants. Most of them are based on the consideration of the radial distance from stations within the local monitoring networks, used as a proxy to the proximity of population subjects within the study groups (56). It should be noted that besides using monitoring data for health impact assessment, modeling method using air quality models is also used to assess HIA.

Since pollutant concentrations in urban environments may vary widely, geostatistical approaches to environmental epidemiology have gained even more relevance (57, 58). The present study aims to present a practical approach to this problem based on the information already gathered in the existing monitoring stations in the Mexico City metropolitan area.

It is expected that data-centered studies, such as this one, will motivate public policy makers to strengthen the monitoring, data gathering and data analysis strategies in large urban environments, such as the metropolitan area of Mexico City.

We are aware of the many challenges that effective environmental assessment has, from economic, logistic and political, but also for technical and analytical reasons. However, we also believe that there are good reasons to be confident that this kind of studies will be relevant for the construction of new, more efficient models of policy making.

## Data Availability Statement

All datasets generated for this study are included in the article/Supplementary Material.

## Author Contributions

KB-L, CF, JE-E, and EH-L made a substantial contributions to conceptualization and methodology, investigation and validation. KB-L, CF, JE-E, MM-G, MC-L, MF-M, and EH-L were involved in the formal analysis and agreed to be accountable for all aspects of the work in ensuring that questions related to the accuracy or integrity of any part of the work are appropriately investigated and resolved, and also were involved in drafting the manuscript or revising it critically for important intellectual content. All authors read and approved the final manuscript.

## Conflict of Interest

The authors declare that the research was conducted in the absence of any commercial or financial relationships that could be construed as a potential conflict of interest.
