# Supplementary material for: Spatio-Temporal Representativeness of Air Quality Monitoring Stations in Mexico City: Implications for Public Health
Source: Front Public Health. 2021 Jan 12;8:536174. doi: 10.3389/fpubh.2020.536174 (PMC7874227; doi:10.3389/fpubh.2020.536174)
Supplement: Supplementary file 1 [file Data_Sheet_1.pdf]

Supplementary Materials: Spatio-Temporal Representativeness of Air Quality Monitoring Stations in Mexico City

Karol Baca-López [1,2,3†], Cristóbal Fresno [4†], Jesús Espinal-Enríquez [3], Miguel Ángel Camacho-López [1], Miriam V. Flores-Merino [5], and Enrique Hernández-Lemus [3,6*]

1. Supplementary tables

Monitoring stations distributed in the Valley of Mexico Metropolitan Area (VMMA).

Table S1. Location coordinates for monitoring stations in the Valley of Mexico Metropolitan Area (VMMA).

	Station ID	Station code	Station name	Longitude	Latitude	Altitude	Comment
1	484150020109	ACO	Acolman	-98.912003	19.635501	2198	
2	484090120609	AJM	Ajusco Medio	-99.207744	19.272161	2548	
3	484150130101	ATI	Atizapan	-99.254133	19.576963	2341	
4	484090140201	BJU	Benito Juárez	-99.159596	19.370464	2249	Ended in 2005
5	484090020301	CAM	Camarones	-99.169794	19.468404	2233	
6	484090030501	CCA	Centro de Ciencias de la Atmósfera	-99.176111	19.326111	2294	
7	484150250109	CHO	Chalco	-98.886088	19.266948	2253	
8	484090040109	CUA	Cuajimalpa	-99.291705	19.365313	2704	
9	484150570109	FAC	FES Acatlán	-99.243524	19.482473	2299	
10	484090150409	HGM	Hospital General de México	-99.152207	19.411617	2234	
11	484090050209	IMP	Inst. Mexicano del Petróleo	-99.147294	19.487561	2250	Ended in 2010
12	484150620109	INN	Investigaciones Nucleares	-99.38052	19.291968	3082	
13	484090060101	IZT	Iztacalco	-99.117641	19.384413	2238	
14	484090150101	LAG	Lagunilla	-99.135183	19.44242	2223	Ended in 2010
15	484150330201	LLA	Los Laureles	-99.039644	19.578792	2230	
16	484151040203	LPR	La Presa	-99.11772	19.534727	2302	
17	484090170127	MER	Merced	-99.119594	19.42.461	2245	
18	484090160609	MGH	Mguel Hidalgo	-99.20266	19.40405	2327	
19	484150990113	MON	Montecillo	-98.902853	19.460415	2252	
20	484090090104	MPA	Milpa Alta	-98.990189	19.1769	2594	
21	484150580115	NEZ	Nezahualcóyotl	-99.028212	19.393734	2235	
22	484090100127	PED	Pedregal	-99.204136	19.325146	2326	
23	484090100209	PLA	Plateros	-99.200109	19.365869	2345	Ended in 2010
24	484150330327	SAG	San Agustín	-99.030324	19.532968	2241	
25	484090040309	SFE	Santa fe	-99.262865	19.357357	2599	
26	484090050701	SJA	San Juan Aragón	-99.086095	19.452592	2258	
27	484090030109	SUR	Santa Ursula	-99.149994	19.31448	2279	
28	484090160309	TAC	Tacuba	-99.202455	19.453907	2275	Ended in 2010
29	484090130309	TAH	Tlahuac	-99.010564	19.246459	2297	
30	484090030201	TAX	Taxqueña	-99.123204	19.335689	2242	Ended in 2010
31	484151040115	TLA	Tlalnepantla	-99.204597	19.529077	2311	
32	484151090101	TLI	Tultitlán	-99.177173	19.602542	2313	

Station ID	Station code	Station name	Longitude	Latitude	Altitude	Comment	
33	484090120209	TPN	Tlalpan	-99.184177	19.257041	2522	
34	484090030401	UAX	UAM Xochimilco	-99.103629	19.304441	2246	
35	484090070219	UIZ	UAM Iztapalapa	-99.07388	19.360794	2221	
36	484090050501	VAL	Vallejo	-99.165702	19.522437	2248	Ended in 2010
37	484150200109	VIF	Villa de las Flores	-99.09659	19.658223	2242	
38	484150330415	XAL	Xalostoc	-99.0824	19.525995	2160	
39	484090020201	AZC	Azcapotzalco	-99.198657	19.487728	2279	Ended in 2010
40	484090070111	CES	Cerro de la Estrella	-99.074678	19.334731	2219	Ended in 2010
41	484090030303	COY	Coyoacán	-99.157101	19.350258	2260	
42	484150950109	CUT	Cuautitlán	-99.198602	19.722186	2263	
43	484090120400	AJU	Ajusco	-99.162611	19.154286	2942	
44	484090050809	GAM	Gustavo A. Madero	-99.094517	19.4827	2227	
45	484090050101	LVI	La Villa	-99.117749	19.46789	2228	Ended in 2010
46	484150580303	PER	La Perla	-98.991858	19.38286	2237	Ended in 2011
47	484090050301	ARA	Aragón	-99.074549	19.470218	2200	Ended in 2010
48	484800150584	FAR	FES Aragón	-99.046176	19.473692	2230	
49	484800090073	SAC	Santiago Acahualtepec	-99.009381	19.34561	2293	

Real time and historical data for pollutant concentrations and meteorological variables can be downloaded using the R package aire.zmvm for active and inactive stations.

Spatio-temporal modeling

Table S2. Initial variogram parameter values for air pollutants.

		**Time**	**Space**	**Joint**		**Time**	**Space**	**Joint**
CO					NO_2			
	nugget	0.06	0.03	0.04		30.08	27.07	28.57
	sill	0.07	0.07	0.07		51.09	59.95	55.52
	range	12.00	20.73	16.36		12.00	20.04	16.02
	stAni	0.20	0.20	0.20		0.30	0.30	0.30
O_3					SO_2			
	nugget	29.36	44.52	36.94		7.30	7.29	7.30
	sill	64.54	55.29	59.91		10.60	10.94	10.77
	range	12.00	21.40	16.70		12.00	21.40	16.70
	stAni	1.31	1.31	1.31		0.90	0.90	0.90
$PM_{2.5}$					PM_{10}			
	nugget	26.79	41.57	34.18		151.42	101.85	126.63
	sill	56.65	47.81	52.23		264.57	264.96	264.76
	range	12.00	20.73	16.36		12.00	21.40	16.70
	stAni	1.49	1.49	1.49		0.51	0.51	0.51
NO					NO_X			
	nugget	91.85	53.94	72.90		178.67	125.15	151.91
	sill	134.66	143.15	138.90		288.20	336.27	312.24
	range	12.00	20.04	16.02		12.00	20.04	16.02
	stAni	0.17	0.17	0.17		0.21	0.21	0.21
PM_{CO}								
	nugget	55.79	42.85	49.32				
	sill	86.17	96.60	91.38				
	range	12.00	15.15	13.57				
	stAni	0.23	0.23	0.23				

stAni: spatio-temporal anisotropy

Table S3. Weighted Mean Squared Error (wMSE) for the tested covariance models.

		Variogram model type: temporal + spatial								
**Model**	**Joint**	**Exp+Exp**	**Sph+Sph**	**Gau+Gau**	**Exp+Gau**	**Exp+Sph**	**Gau+Exp**	**Gau+Sph**	**Sph+Exp**	**Sph+Gau**
					**CO** ($x10^{-5}$)					
metric		12.687465	13.755002	13109554						
separable		15.934256	16.067906	15.769925	17.658642	17.667964	17.524822	19.864663	19.844149	20.010841
productSum		10.387669	10.701669	10.567512	11.069849	11.383828	11.249604	12.022900	12.336960	12.202713
sumMetric	Exp	9.1094946	9.3539013	9.3467147	9.1827588	9.1909141	9.1792908	9.2375209	9.2445766	9.2309799
	Gau	9.2477978	**9.0862766**	9.2445509	9.1124273	9.1760317	9.1738422	9.1540216	9.3166022	9.3023450
	Sph	9.5790768	9.5980817	9.7209405	9.9303779	9.9290617	10.056813	10.306468	10.362968	10.478897
simpleSumMetric	Exp	10.930088	11.648282	11.189374	11.782681	12.038947	11.180347	11.054112	11.917533	10.994724
	Gau	11.428389	12.246122	11.434344	11.408763	12.496029	11.346504	11.276083	12.335031	11.203231
	Sph	10.942809	11.343366	11.084135	11.529140	11.920329	11.671441	11.699319	12.056540	11.840963
					**NO_2**					
metric		36.243773	36.190178	37.335321						
separable		19.739679	18.730610	28.301287	25.021973	20.785831	21.323688	20.197042	18.872294	64.086134
productSum		157.39009	159.61802	104.37028	157.85534	160.22477	157.98531	152.66819	156.47556	150.22468
sumMetric	Exp	20.405467	19.356675	19.653267	20.974543	19.547023	19.540268	21.124485	21.384224	19.653936
	Gau	18.678240	18.850684	18.875713	19.255862	19.411635	19.022950	**18.621923**	18.828260	18.879197
	Sph	19.915800	19.926116	19.563619	20.825363	20.611311	20.590186	21.024601	20.985965	20.359239
simpleSumMetric	Exp	21.528590	22.915373	23.663630	20.517779	27.712893	27.978688	20.922169	24.832197	19.918765
	Gau	21.145796	24.379904	20.472094	20.503843	25.145816	20.464708	20.502659	25.134281	20.472188
	Sph	23.262429	2.2988019	2.3442219	2.0663130	2.7760479	2.8136305	2.3320050	2.6415097	2.0119241
					**O_3**					
metric		36.738531	37.516546	40.169483						
separable		12.862907	12.137729	12.1804905	16.551945	15.811119	16.384620	12.820999	12.051422	13.05328
productSum		120.04941	131.77844	131.90146	132.38114	132.47841	132.36235	130.93283	125.40686	131.78258
sumMetric	Exp	12.959113	12.526391	12.828179	12.808789	12.036874	12.665899	12.914518	12.397388	12.780444
	Gau	12.523563	12.186858	12.397406	13.329433	13.359219	13.810981	12.832936	12.649313	12.661316
	Sph	12.786608	12.071838	12.733775	12.586287	**11.739013**	12.384449	12.921369	12.028041	12.722199
simpleSumMetric	Exp	14.248095	22.474226	14.257071	14.857464	16.287031	14.885195	24.646847	24.618915	24.624416
	Gau	15.540192	16.731454	16.031641	15.471850	17.241622	15.272079	24.602046	17.269955	24.631240
	Sph	14.265159	15.662118	14.276679	14.995673	16.386956	15.004850	14.196723	15.595944	14.215573

Table S3. Weighted Mean Squared Error (wMSE) for the tested covariance models.

		Variogram model type: temporal + spatial								
**Model**	**Joint**	**Exp+Exp**	**Sph+Sph**	**Gau+Gau**	**Exp+Gau**	**Exp+Sph**	**Gau+Exp**	**Gau+Sph**	**Sph+Exp**	**Sph+Gau**
					**SO_2**					
metric		2.2971602	2.9276259	2.3165600						
separable		1.2973798	1.4829583	1.3365423	1.3250730	1.5104900	1.4788553	1.4582871	1.4855978	1.3384686
productSum		1.4409721	1.8550175	1.4809262	1.4640537	1.9126299	1.4179524	1.3684402	1.9855293	1.3541143
sumMetric	Exp	1.1467001	1.1446639	1.1603865	1.1582012	1.1508205	1.1366746	1.1689694	1.1576013	1.1491171
	Gau	1.1905485	1.1074646	1.1592508	1.1863640	1.0981134	1.1694991	1.1858093	1.1199713	1.1527094
	Sph	1.1414655	1.1468795	1.1563712	1.1357774	1.1002742	1.1683315	1.1481389	1.1497978	1.1799877
simpleSumMetric	Exp	1.1690903	1.2498606	1.1755777	1.1693680	1.2785529	1.1760391	1.1688655	1.2785525	1.1495043
	Gau	1.1911346	**1.0825454**	1.1671219	1.1871278	1.2349213	1.1643071	1.1871409	1.2351152	1.1643210
	Sph	1.1781904	1.0867948	1.1583632	1.3965945	1.1019733	1.3529013	1.2588179	1.2429075	1.2629365
					**$PM_{2.5}$**					
metric		39.922897	42.345209	46.754791						
separable		28.507500	29.401127	28.591856	29.662620	30.182863	29.744086	29.631128	30.209324	29.709811
productSum		105.43910	106.89133	106.72626	107.27106	107.32361	107.22165	106.94987	106.14729	106.47879
sumMetric	Exp	28.194741	29.057137	28.307729	28.317976	28.737220	28.386897	27.791192	28.912476	27.852497
	Gau	28.305894	26.471481	28.457846	26.141677	25.847649	26.218916	28.076820	28.708826	28.110590
	Sph	26.483316	29.155068	26.421350	25.720645	28.550828	27.474676	25.906585	26.156186	27.653929
simpleSumMetric	Exp	28.685693	29.654383	29.128327	25.735200	25.551780	**25.525934**	28.298600	31.041913	28.569461
	Gau	28.316387	30.536618	28.811262	28.606974	30.713249	28.729853	34.842684	38.823842	34.290173
	Sph	26.479810	26.279685	26.260819	25.719246	25.619594	25.614128	25.900608	25.732411	25.670365
					**PM_{10}** ($x10^3$)					
metric		2.1834637	2.1642924	2.2108578						
separable		1.6726345	1.8826978	1.7364406	1.6887697	1.9317984	1.6935050	1.6585776	1.8840431	1.6471243
productSum		4.9942065	5.0136686	4.9859243	5.0033602	5.0274988	5.0015739	4.9918017	5.0091412	4.9881407
sumMetric	Exp	1.6001574	1.6062182	1.6123121	1.6440699	1.6529180	1.6567818	1.6042075	1.5939756	1.6157839
	Gau	1.6068184	1.5895467	1.6043744	1.6203514	1.6321256	1.6244107	1.6062513	**1.5890730**	1.6050473
	Sph	1.6087894	1.6033047	1.6121965	1.6534245	1.6519912	1.6579449	1.6221581	1.6087212	1.6106236
simpleSumMetric	Exp	1.7088028	1.7029779	1.6697482	1.8332621	1.9250678	1.8718995	1.7224186	1.7383409	1.7192381
	Gau	1.6187367	1.6710570	1.6090375	1.6428340	1.7841496	1.6255546	1.7302014	1.6788229	1.7283864
	Sph	1.6878275	1.7140486	1.7331586	1.8216197	1.9326248	1.8254637	1.7143718	1.7131918	1.7593735

Table S3. Weighted Mean Squared Error (wMSE) for the tested covariance models.

Model	Joint	Exp+Exp	Sph+Sph	Gau+Gau	Exp+Gau	Exp+Sph	Gau+Exp	Gau+Sph	Sph+Exp	Sph+Gau
		**Variogram model type: temporal + spatial**								
						**NO**				
metric		314.32251	464.49668	320.77210						
separable		227.29274	267.19351	223.27802	218.93799	227.45143	228.41019	332.39790	238.98087	224.69833
productSum		998.05200	1003.7673	1004.2407	997.54517	1006.2720	999.47112	1007.2452	1019.3748	1004.1995
sumMetric	Exp	223.65028	229.25265	222.33345	219.14968	219.93966	**217.79853**	221.95089	237.60784	221.03913
	Gau	218.41711	228.49110	219.51056	220.07645	221.93173	220.38515	219.47318	223.67573	219.12299
	Sph	224.55070	223.98598	224.78247	218.43059	219.30839	218.53857	221.33537	223.16202	220.57676
simpleSumMetric	Exp	251.47818	251.84904	251.22780	261.28988	262.41332	260.69236	250.97997	251.47600	251.62466
	Gau	249.67680	289.37639	254.66438	250.05371	275.91811	263.48996	252.56001	291.14505	255.62076
	Sph	251.37048	255.90646	255.02151	260.57654	265.68344	267.31847	251.58737	255.22089	254.95225
						**NO_X**				
metric		1216.2399	1215.8008	1255.7962						
separable		783.89513	823.63824	924.78019	782.54520	809.71588	770.71427	781.20906	1319.5791	815.85242
productSum		5500.8853	5534.3305	5508.4015	5515.9607	5554.1369	5512.5675	5483.0362	5521.7044	5473.9558
sumMetric	Exp	811.09989	852.74722	773.49901	807.50987	1028.0700	762.12053	796.50774	965.96022	782.51881
	Gau	762.32740	797.58761	**756.12147**	761.97337	800.26970	763.33415	772.35853	790.17162	763.07860
	Sph	785.29970	794.28077	777.47023	775.57787	783.13504	770.92203	809.17473	780.19179	770.48375
simpleSumMetric	Exp	901.63339	894.10510	897.27721	980.04353	993.26101	968.35391	902.79395	897.97592	899.07844
	Gau	889.57147	1076.5107	901.19040	820.13383	1145.2760	931.71713	894.79796	1040.1277	899.95515
	Sph	897.35755	905.11647	898.36671	969.51013	993.98581	970.00722	899.30342	903.66063	900.75456
						**PM_{CO}**				
metric		644.62111	644.03842	643.45868						
separable		623.71605	607.71669	610.39054	616.96630	604.77784	614.93006	612.03043	632.08639	793.09965
productSum		950.07066	951.46203	942.46674	951.04700	952.21029	944.12622	1061.7258	942.02212	895.38683
sumMetric	Exp	609.97906	609.29677	607.78059	614.03327	614.83170	611.33476	610.85005	611.61747	608.65790
	Gau	603.57826	599.57778	602.80172	606.53313	603.43250	603.50454	601.47158	**596.46374**	600.59666
	Sph	607.76432	608.52111	606.79730	612.11267	612.54121	611.14899	608.07712	608.12503	607.45357
simpleSumMetric	Exp	608.83922	612.20342	606.55306	612.76328	627.74877	606.69545	610.48604	609.94741	606.39853
	Gau	603.55459	607.78308	603.88012	605.75264	609.40062	604.36625	603.01894	607.90093	602.39252
	Sph	606.93907	606.86581	612.94685	613.81471	616.68362	633.30340	609.15736	612.67814	607.15921

Table S4. Final variogram parameter values for air pollutants.

	Time	Space	Joint		Time	Space	Joint
CO				NO_2			
		sumMetric				sumMetric	
	Exp	Gau	Gau		Sph	Exp	Gau
nugget	0.00988	0.02448	0.00597		9.83256	6.04587	0.14808
sill	0.00715	0.00100	0.02858		8.32812	48.98035	59.93512
range	12.00172	20.72637	16.36296		27.48596	43.37174	34.37637
stAni			0.32459				0.31863
O_3				SO_2			
		sumMetric				simpleSumMetric	
	Gau	Gau	Sph		Gau	Exp	Gau
nugget	17.39906	8.17109	0.997322				0.19818
sill	56.57224	60.82626	12.19842		2.29675	3.35601	5.96559
range	73.3575	57.82687	97.79145		37.47534	5.92587	35.92549
stAni			3.61389				25.58136
$PM_{2.5}$				PM_{10}			
		simpleSumMetric				sumMetric	
	Gau	Sph	Exp		Sph	Gau	Gau
nugget			3.84173		61.16633	0.0010	13.48901
sill	26.15424	9.60075	22.41689		172.35015	155.2557	246.47684
range	36.65058	13.0008	128.6212		178.9469	12.9828	176.4812
stAni			65.67073				0.63066
NO				NO_X			
		sumMetric				sumMetric	
	Gau	Sph	Exp		Exp	Sph	Gau
nugget	23.37486	23.82696	4.82430		46.04706	50.46123	16.25752
sill	107.66649	171.11593	149.16515		230.64259	338.27802	317.65596
range	133.497	77.15998	123.2927		175.6062	84.44737	38.76529
stAni			0.39323				0.24669
PM_{CO}							
		sumMetric					
	Sph	Gau	Gau				
nugget	8.15818	13.60533	9.42787				
sill	18.96224	98.58390	93.12793				
range	21.24231	28.04363	33.89663				
stAni			0.25065				

stAni: spatio-temporal anisotropy

2. Supplementary figures

Data capture per contaminant and monitoring station

Sample variogram and fitted covariance models

The winner permutations taken by one, two or three variograms (Exponential, Gaussian and/or Spherical) for independent spatio, temporal and joint spatio-temporal models according to the covariance structure (metric, separable, productSum, sumMetric and simpleSumMetric) are presented for carbon monoxide (CO), nitrogen dioxide (NO_2), ozone (O_3), sulphur dioxide (SO_2), particulate matter ($PM_{2.5}$ and PM_{10}), nitrogen oxide (NO), other nitrogen oxides (NO_X) and coarse particulate matter (PM_{CO}) in Supplementary Figures **??**-**??** respectively.

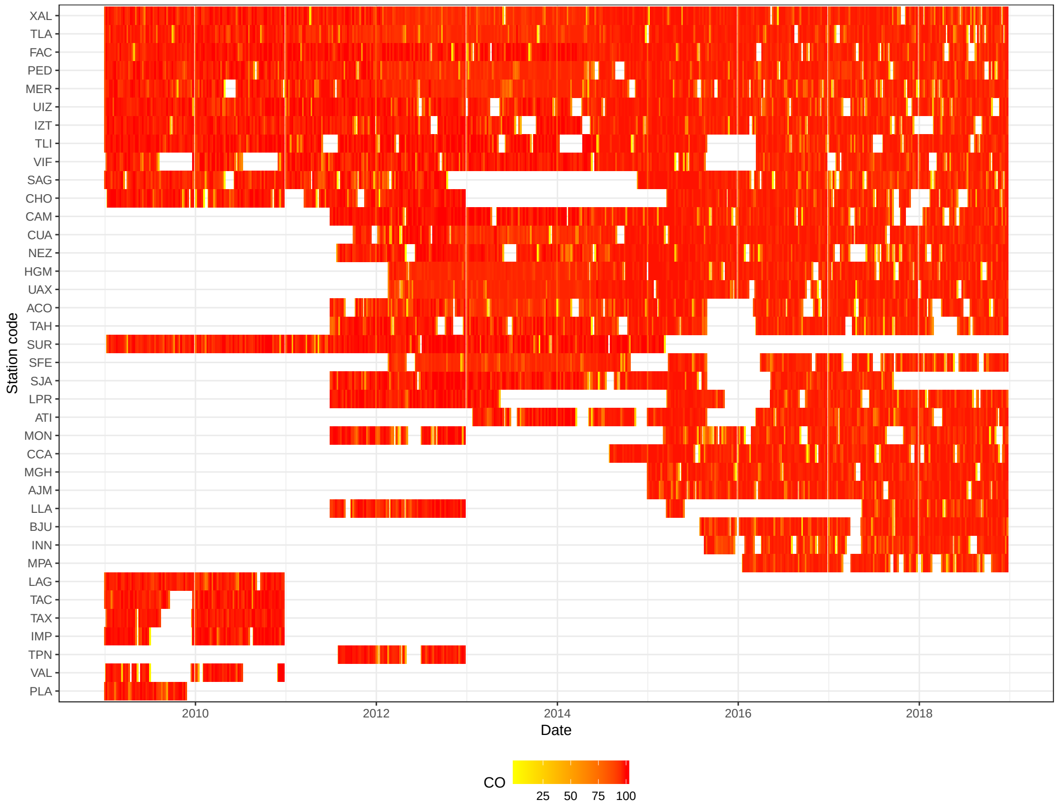

Figure S1. Carbon monoxide (CO) data capture. The color bar indicates the percentage of available data.

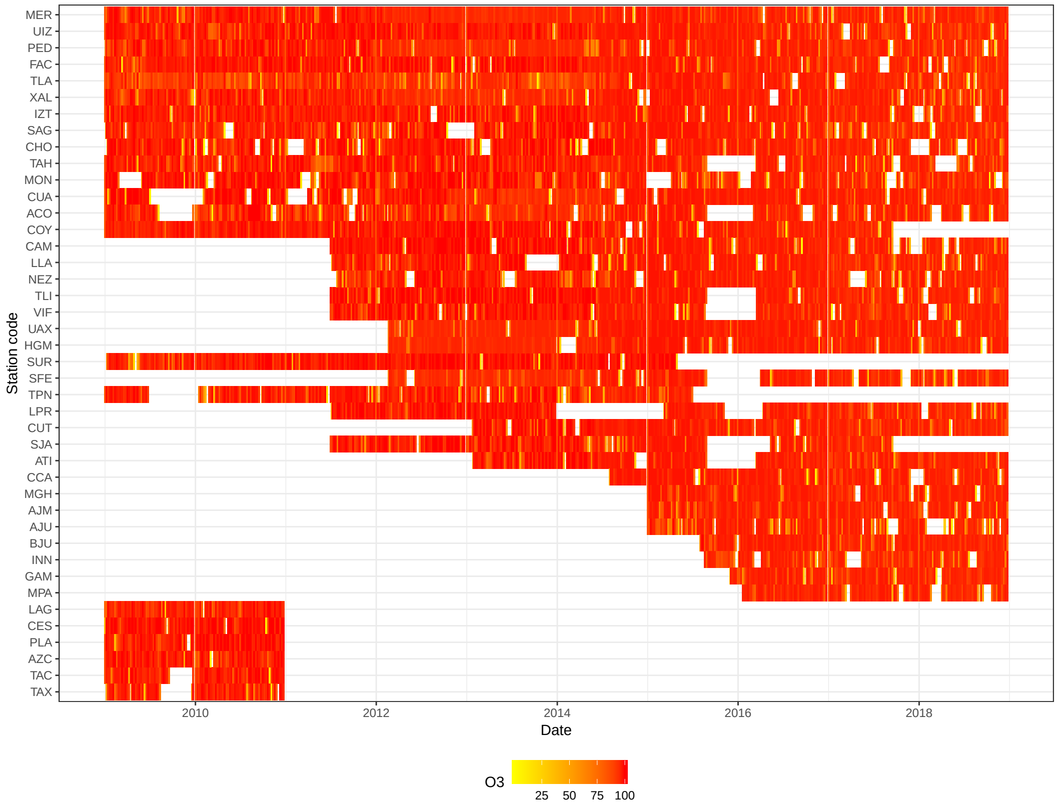

Figure S2. Ozone (O_3) data capture. The color bar indicates the percentage of available data.

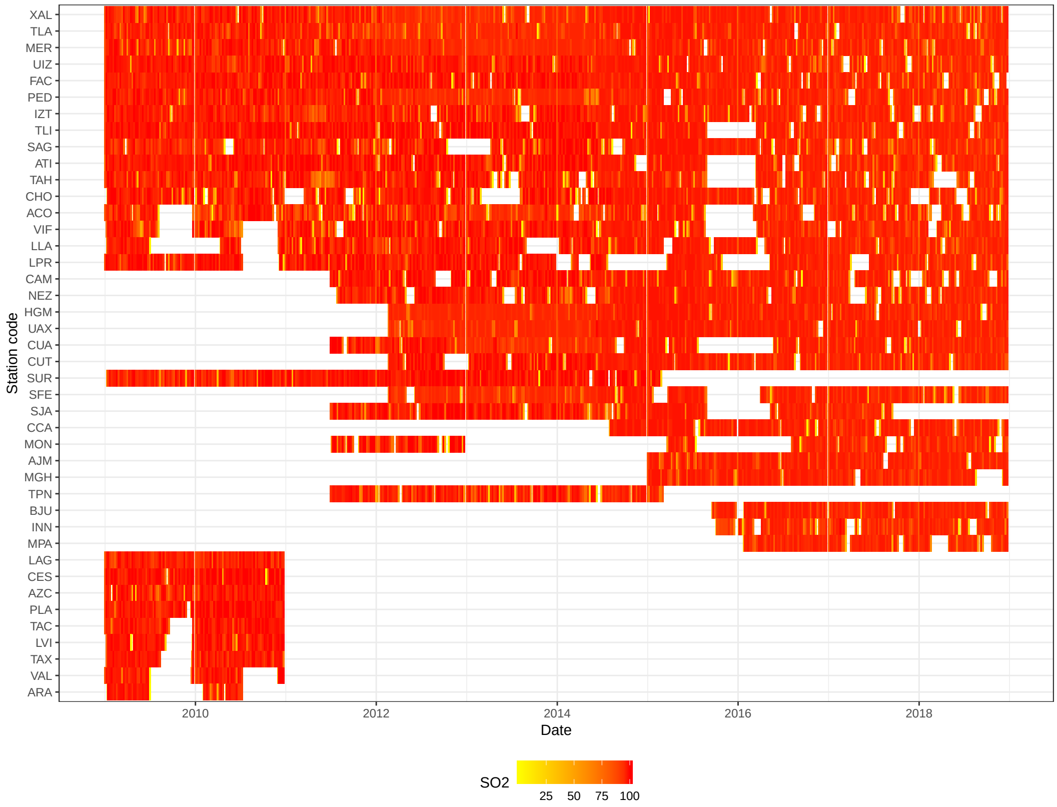

Figure S3. Sulphur dioxide (SO_2) data capture. The color bar indicates the percentage of available data.

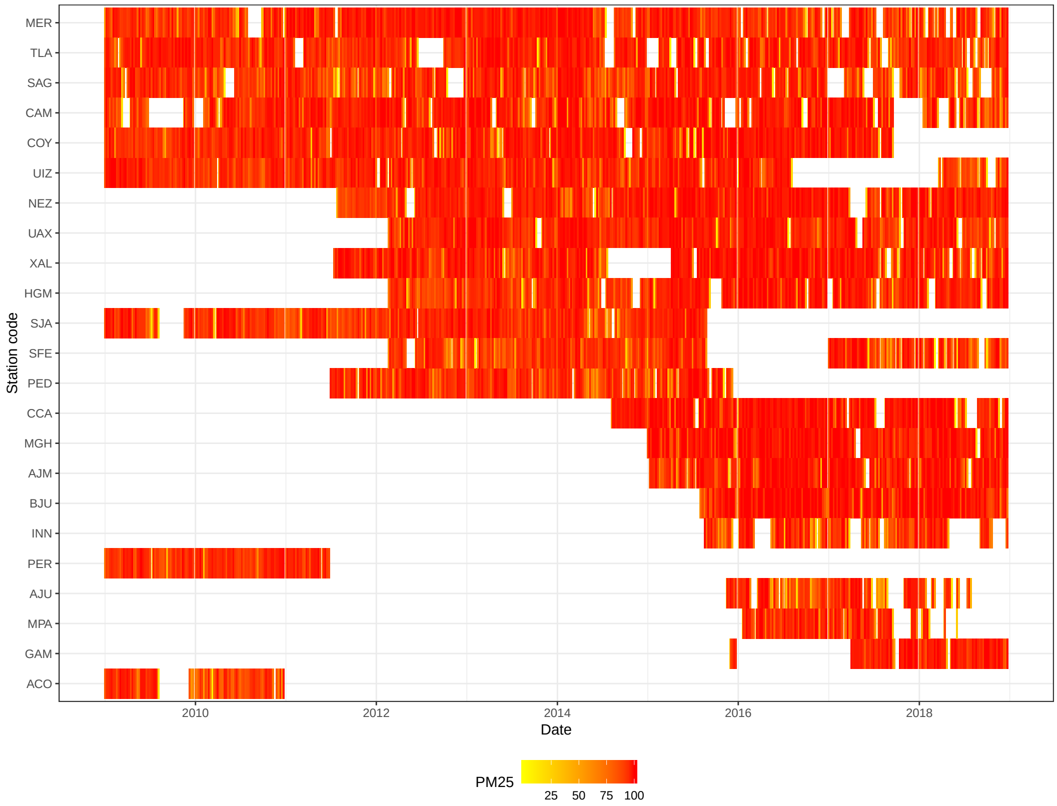

Figure S4. Particulate matter $PM_{2.5}$ data capture. The color bar indicates the percentage of available data.

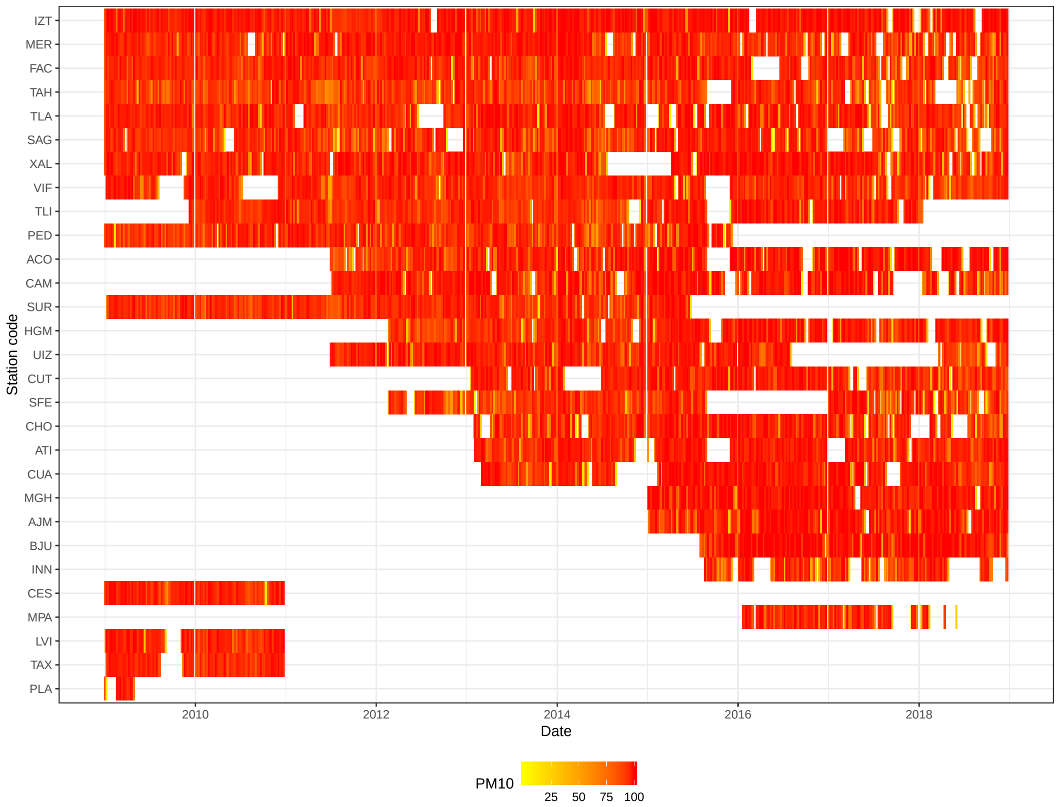

Figure S5. Particulate matter PM_{10} data capture. The color bar indicates the percentage of available data.

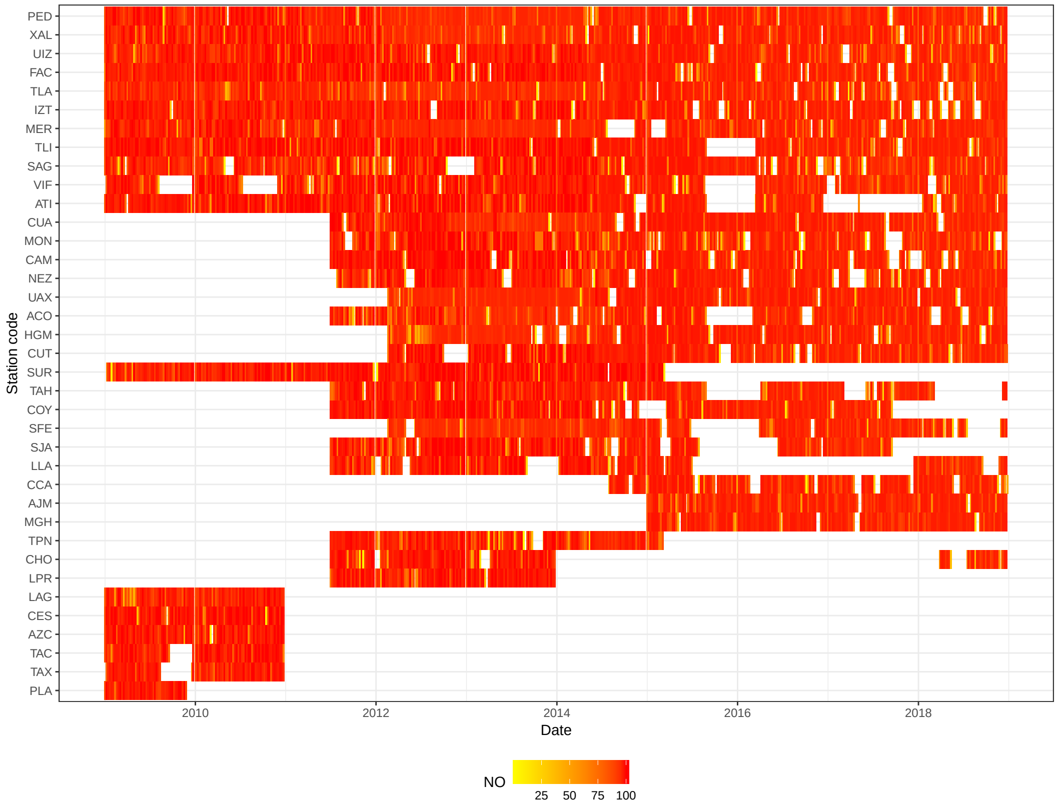

Figure S6. Nitrogen oxide (NO) data capture. The color bar indicates the percentage of available data.

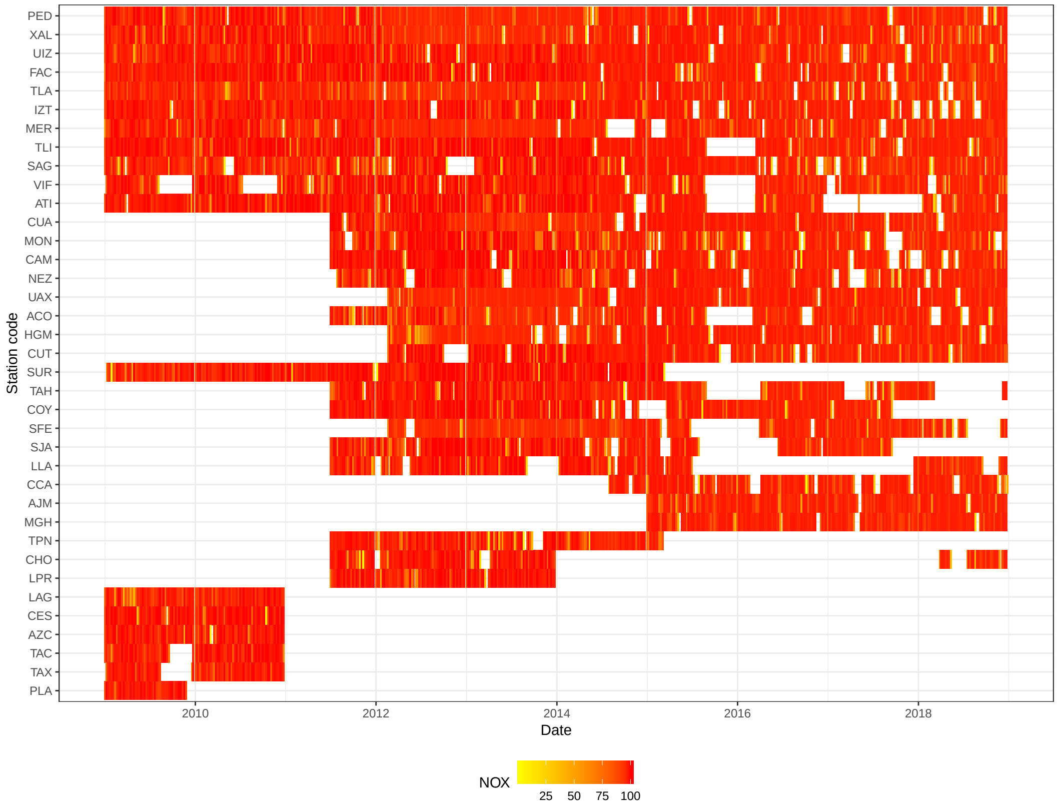

Figure S7. Nitrogen oxides (NO_X) data capture. The color bar indicates the percentage of available data.

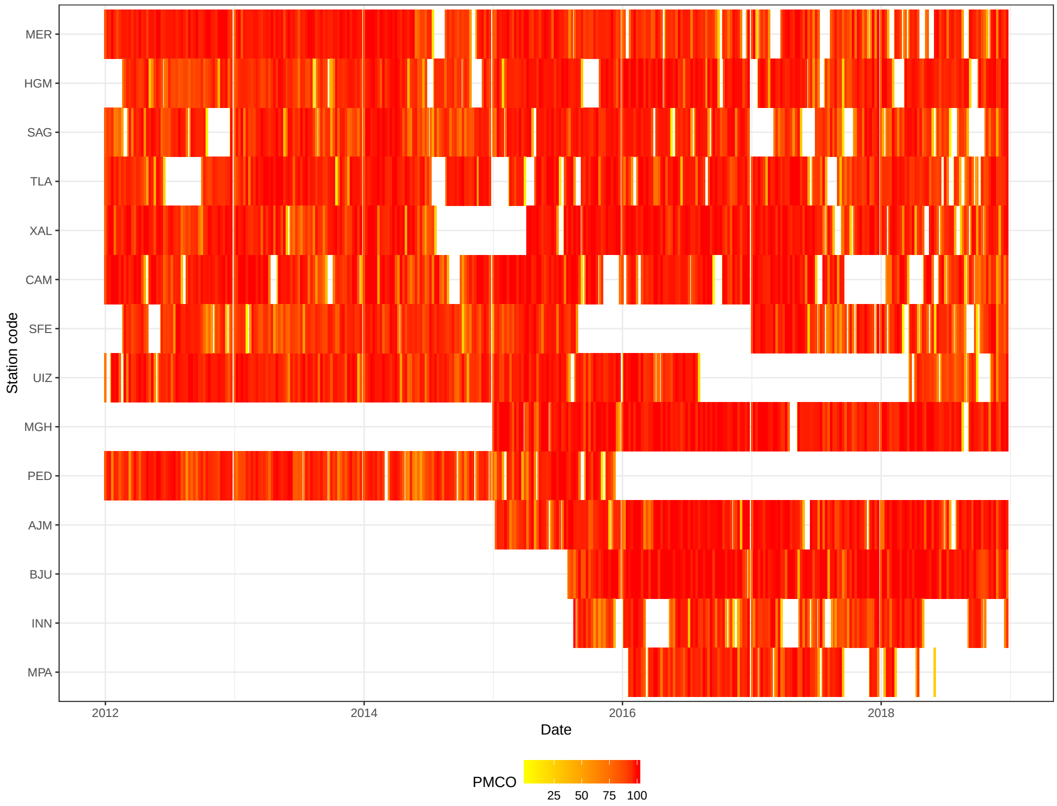

Figure S8. Coarse particulate matter PM_{CO} data capture. The color bar indicates the percentage of available data.

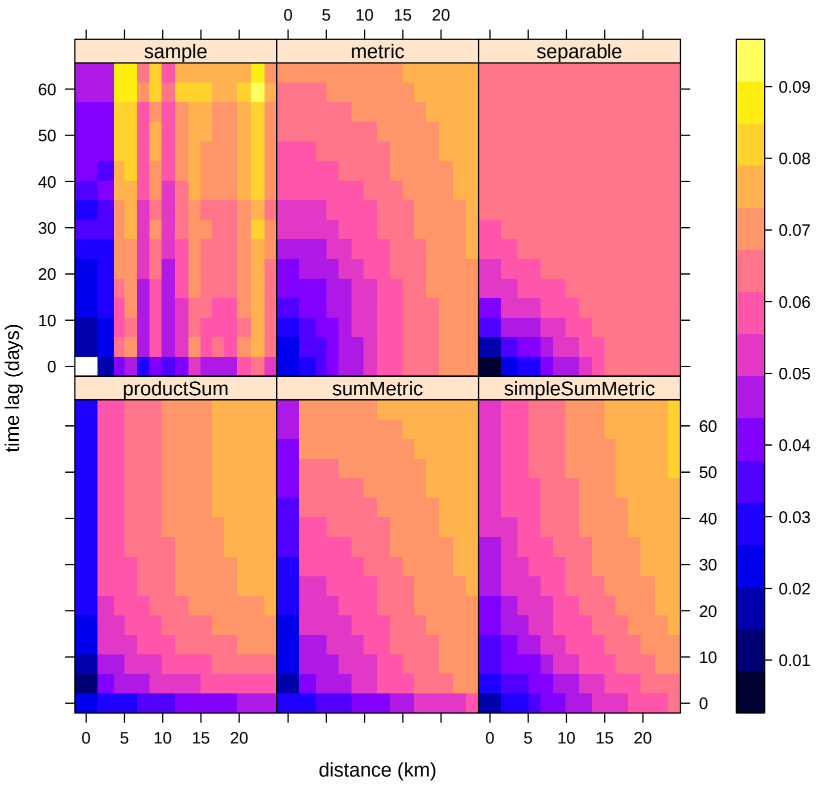

Figure S9. Sample variogram and fitted covariance models for carbon monoxide (CO).

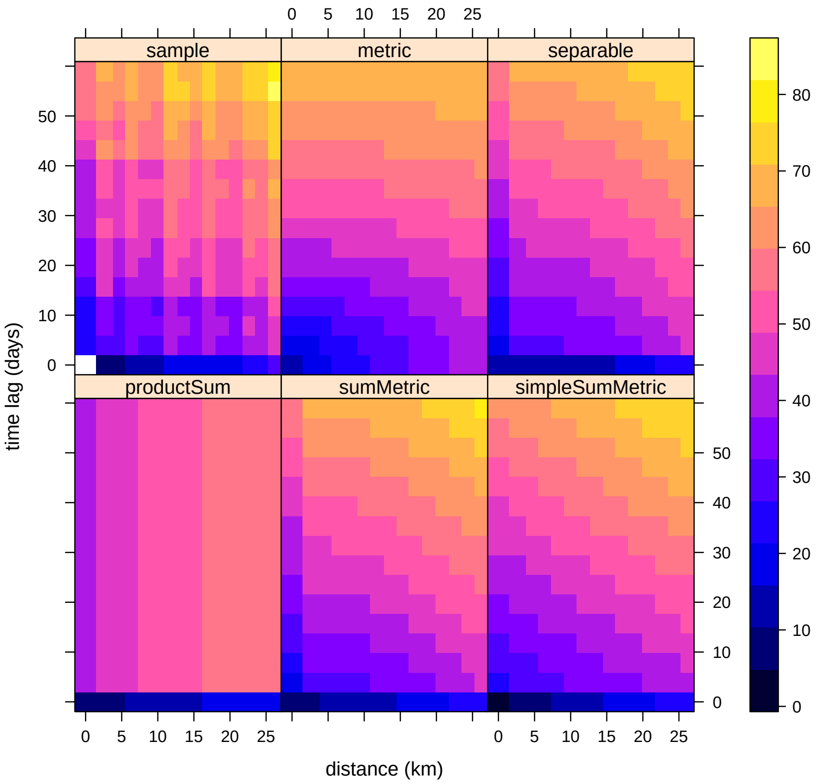

Figure S10. Sample variogram and fitted covariance models for ozone (O_3).

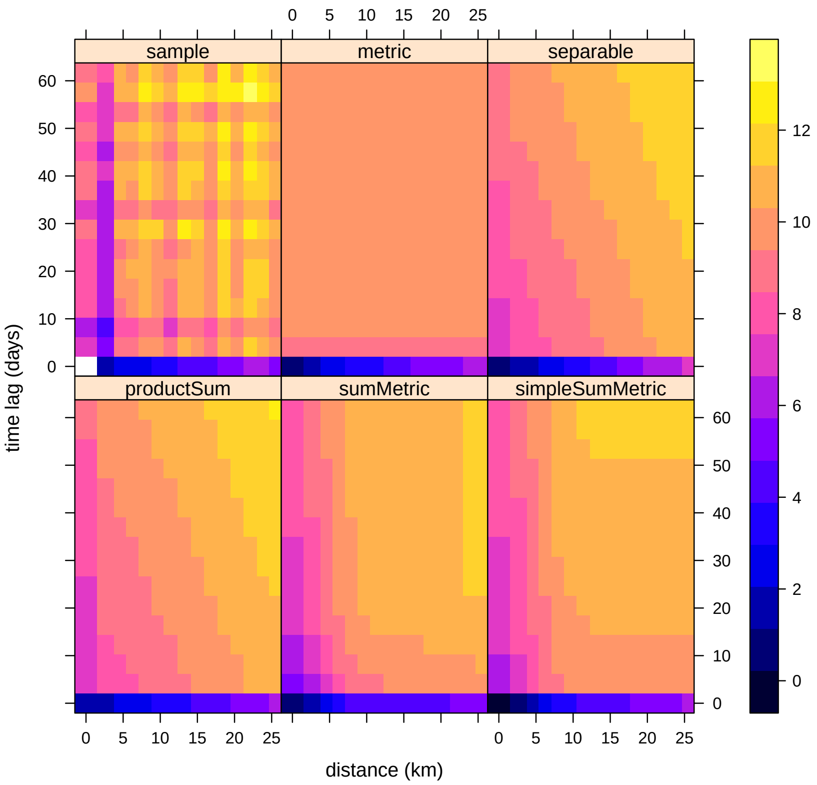

Figure S11. Sample variogram and fitted covariance models for sulphur dioxide (SO_2).

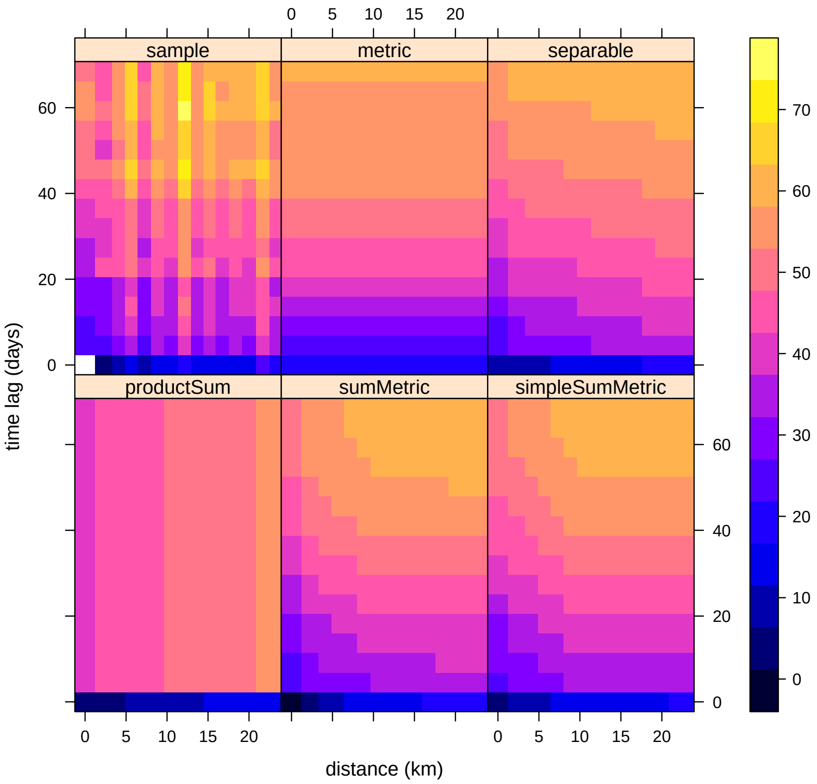

Figure S12. Sample variogram and fitted covariance models for particulate matter $PM_{2.5}$.

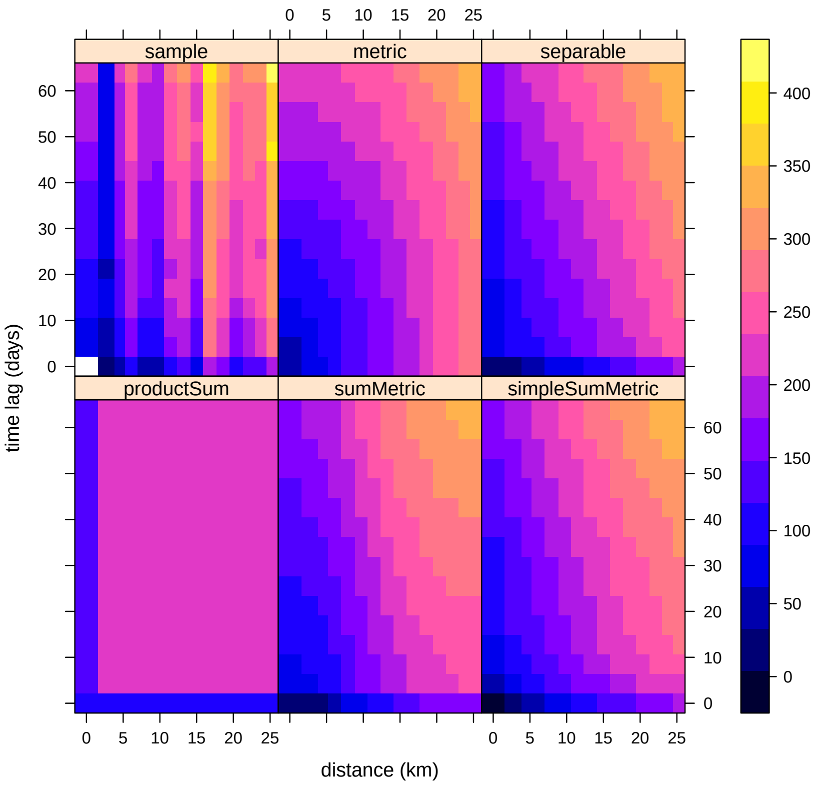

Figure S13. Sample variogram and fitted covariance models for particulate matter PM_{10}.

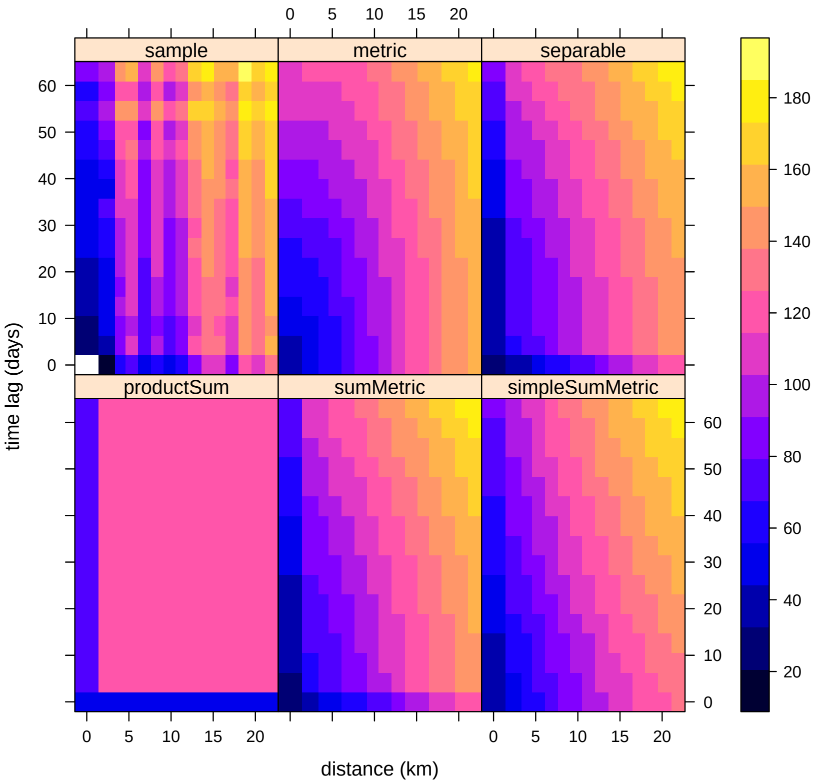

Figure S14. Sample variogram and fitted covariance models for nitrogen oxide (NO).

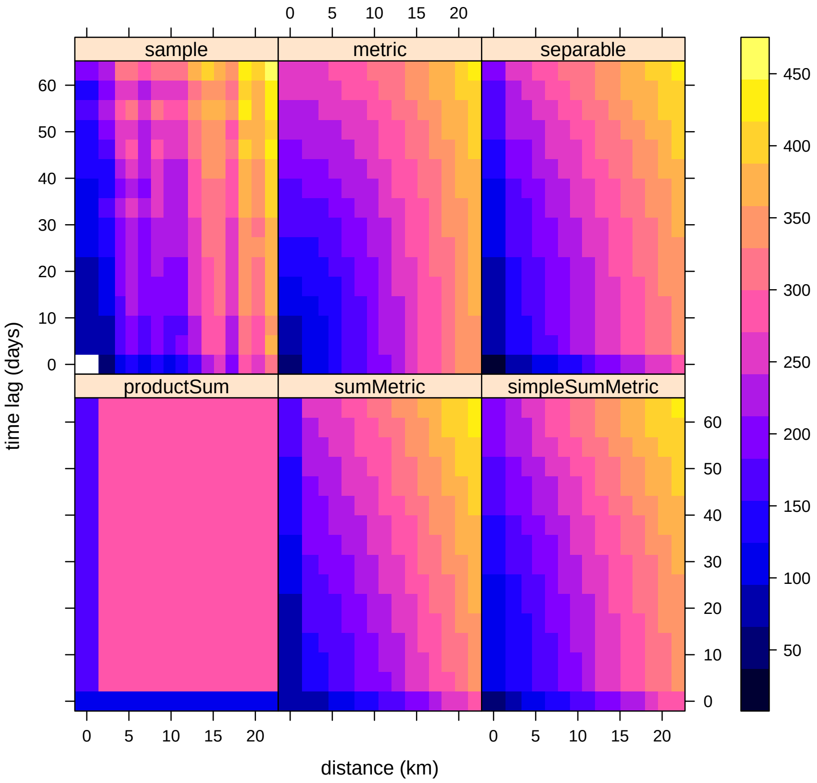

Figure S15. Sample variogram and fitted covariance models for nitrogen oxides (NO_X).

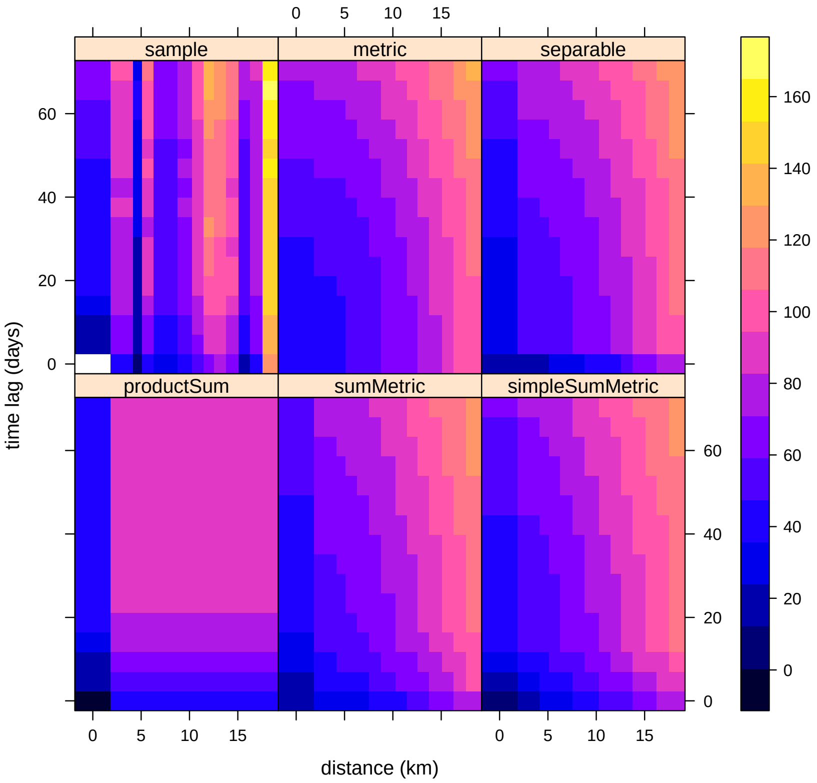

Figure S16. Sample variogram and fitted covariance models for coarse particulate matter (PM_{CO}).

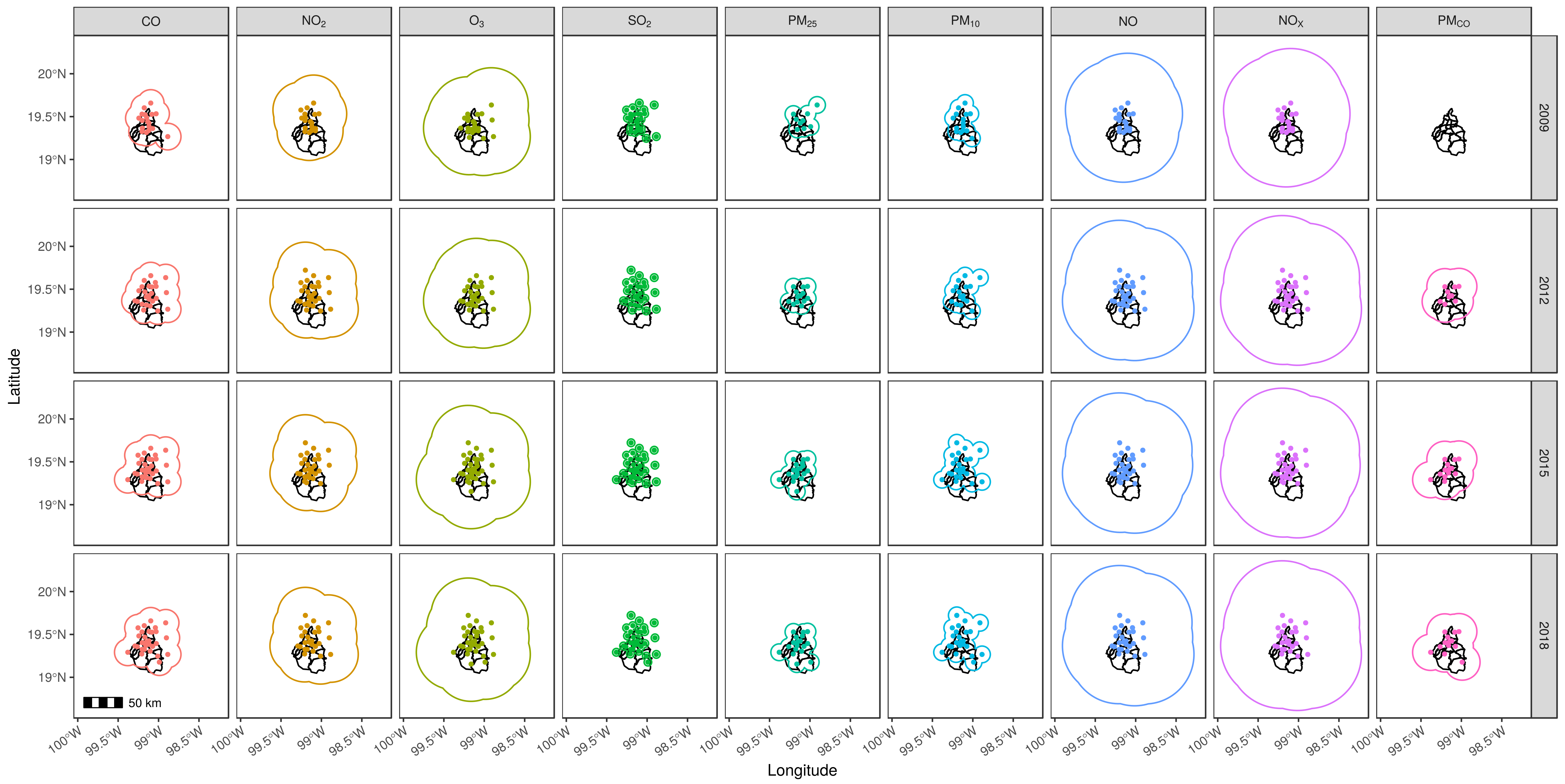

CO
NO$_2$
O$_3$
SO$_2$
PM$_{25}$
PM$_{10}$
NO
NO$_X$
PM$_{CO}$
2009
2012
2015
2018
20°N
19.5°N
19°N
Latitude
100°W
99.5°W
99°W
98.5°W
Longitude
50 km
